# Identification of microRNAs in PCV2 subclinically infected pigs by high throughput sequencing

**DOI:** 10.1186/s13567-014-0141-4

**Published:** 2015-03-03

**Authors:** Fernando Núñez-Hernández, Lester J Pérez, Marta Muñoz, Gonzalo Vera, Anna Tomás, Raquel Egea, Sarai Córdoba, Joaquim Segalés, Armand Sánchez, José I Núñez

**Affiliations:** Centre de Recerca en Sanitat Animal (CReSA), UAB-IRTA, Campus de la Universitat Autònoma de Barcelona, Bellaterra, Cerdanyola del Vallès Spain; Centro Nacional de Sanidad Agropecuaria (CENSA), La Habana, Cuba; Departament de Genètica Animal, Centre de Recerca en AgriGenòmica (CRAG), CSIC- IRTA-UAB-UB, Universitat Autònoma de Barcelona, Bellaterra, Barcelona, Spain; Programa Infección e Inmunidad, Fundació d´Investigació Sanitària de les illes Balears, 07110 Buynola, Spain; Departament de Sanitat i Anatomia Animals, Universitat Autònoma de Barcelona, Bellaterra, Barcelona, Spain; Departament de Ciència Animal i dels Aliments, Universitat Autònoma de Barcelona (UAB), Bellaterra, Barcelona, Spain

## Abstract

Porcine circovirus type 2 (PCV2) is the essential etiological infectious agent of PCV2-systemic disease and has been associated with other swine diseases, all of them collectively known as porcine circovirus diseases. MicroRNAs (miRNAs) are a new class of small non-coding RNAs that regulate gene expression post-transcriptionally. miRNAs play an increasing role in many biological processes. The study of miRNA-mediated host-pathogen interactions has emerged in the last decade due to the important role that miRNAs play in antiviral defense. The objective of this study was to identify the miRNA expression pattern in PCV2 subclinically infected and non-infected pigs. For this purpose an experimental PCV2 infection was carried out and small-RNA libraries were constructed from tonsil and mediastinal lymph node (MLN) of infected and non-infected pigs. High throughput sequencing determined differences in miRNA expression in MLN between infected and non-infected while, in tonsil, a very conserved pattern was observed. In MLN, miRNA 126-3p, miRNA 126-5p, let-7d-3p, mir-129a and mir-let-7b-3p were up-regulated whereas mir-193a-5p, mir-574-5p and mir-34a down-regulated. Prediction of functional analysis showed that these miRNAs can be involved in pathways related to immune system and in processes related to the pathogenesis of PCV2, although functional assays are needed to support these predictions. This is the first study on miRNA gene expression in pigs infected with PCV2 using a high throughput sequencing approach in which several host miRNAs were differentially expressed in response to PCV2 infection.

**Electronic supplementary material:**

The online version of this article (doi:10.1186/s13567-014-0141-4) contains supplementary material, which is available to authorized users.

## Introduction

Porcine circovirus type 2 (PCV2) belongs to the *Circoviridae* family. The viral particle contains a single-strand circular DNA genome of 1768-9 nucleotides (nt), enclosed within a non-enveloped protein capsid with a diameter of 16- 18 nm. PCV2 is one of the smallest mammalian viruses encoding 11 potential reading frames, although expression has only been determined from 3 of them. ORF1 encodes the non-structural replication-associated protein Rep and its truncated variant Rep’ [1], ORF2 encodes the structural capsid protein Cap [2] and a non-structural protein with an uncertain function is encoded by ORF3 [3]. Cap and Rep/Rep’ carry out the two most elementary functions of a virus, copying and the successive packaging of the viral genome [4].

PCV2 is the etiological agent of PCV2-systemic disease (PCV2-SD), formerly known as postweaning multisystemic wasting syndrome, (PMWS) [5], an emerging disease in swine first described in 1991 [6]. PCV2 infection is widespread and its most frequent manifestation is by means of a subclinical infection. PCV2 is ubiquitous in swine livestock worldwide, but it has been demonstrated that PCV2 DNA load in serum is significantly higher in PCV2-SD affected pigs than in healthy pigs, which is considered an indicator of the disease [7]. PCV2-SD has a relatively high fatality rate among 5 to 12-week-old pigs. The disease from a clinical point of view causes dyspnea, a progressive loss of weight, anemia, tachypnea, diarrhea and jaundice. Microscopic lesions include lymphadenopathy, nephritis, pancreatitis, hepatitis and granulomatous interstitial pneumonia [6]. PCV2 is thought to be involved in the pathogenesis of porcine dermatitis and nephropathy syndrome (PDNS), and is linked with the occurrence of reproductive disease [5].

It has been suggested that PCV2 replicates firstly in the tonsil and in the regional lymph nodes [6]. PCV2 pathogenesis is related to the immunosuppression caused by the virus in pigs [8] and changes in cytokine production can play a role in this immunosuppression. Pigs with naturally acquired PCV2-SD had an altered cytokine mRNA expression pattern with overexpression of IL-10 mRNA in thymus and IFN-γ mRNA in tonsil, whereas a reduction in the expression of IFN-γ, IL-10, IL-12p40, IL-4 and IL-2 mRNA was observed in other lymphoid tissues [9]. Nevertheless, the mechanisms involved in these processes are poorly understood. This complexity is reflected, for example, in IL-10 expression where a feedback regulation between IL-10 and several microRNAs (miRNAs) has been described [10].

miRNAs are 19-24 nt long non-coding ssRNAs that regulate gene expression post-transcriptionally. Derived from hairpin precursors, they mediate the post-transcriptional silencing of an estimated 30% of protein coding genes in mammals by binding to complementary sites typically located in the 3′ untranslated regions (UTRs) of their target mRNAs [11,12]. This regulation of gene expression via microRNA-mediated RNA interference (RNAi) was first identified in *Caenorhabditis elegans* in 1998. Since this time, more than 21 000 miRNAs have been identified in all kind of species (miRNA registry at miRBase [13]) from mammals, to plants [14], and more recently in several viruses [15,16]. miRNAs have been shown to be implicated in several biological processes such as development, differentiation, homeostasis, carcinogenesis and a wide variety of diseases [17].

The biogenesis of miRNAs has been extensively described in the literature [12,18], but it is considered that there is a canonical pathway by which miRNAs are generated through consecutive cleavages of hairpin precursors by two RNase III enzymes, Drosha and Dicer. In the cell nucleus, the single strand-double strand junction of the pri-miRNA hairpin is recognized by Pasha (or DGCR8 in vertebrates), which is essential for the RNase III enzyme Drosha processing of the hairpin structure. This cleavage generates a 55–70 pre-miRNA hairpin that is exported to the cytoplasm assisted by exportin-5. Once in the cytoplasm, the terminal loop is split from the structure by the RNase III enzyme Dicer. Then, the duplexes are unwound to load the mature strand into an Argonaute containing RNA-induced silencing complex (RISC) [19,20]. The first 2-8 nucleotides (seed) of the miRNAs are essential in the recognition of the target, the complementarity can be complete or partial, and leads to mRNA translational repression or destabilization.

In this paper, the expression of microRNAs in subclinically PCV2 infected pigs was analysed using high throughput sequencing.

## Material and methods

### Animal infection

Six 6-week-old Landrace x Large White pigs were used. Four of them were intranasally inoculated with a total dose of 7 × 10^4.8^TCID_50_ of PCV2 genotype b isolate Sp-10-7-54-13 [21], while 2 control pigs received PBS by the same route. At 21 days post-inoculation (dpi), animals were euthanized. Samples from eight tissues: spleen, inguinal lymph node, kidney, tonsil, thymus, mediastinal lymph node (MLN), lung and mesenterical lymph node, were collected in duplicate and immediately frozen in liquid nitrogen and stored at −80 °C. A third sample was collected in formalin for histopathological studies. Tissue sections were stained with haematoxylin and eosin, and in situ hybridization (ISH) was carried out in order to detect viral genome.

All animal experiments were performed in the CReSA facilities, and all procedures were carried out according to the guidelines of the institutional animal ethics committee of UAB, preserving the Spanish and European animal experimentation ethics law.

### Homogenate and DNA- RNA extraction

Tissue samples of approximately 100 mg were homogenated with a pestle in 1 mL of Trizol (Invitrogen, Carlsbad, USA) to lyse the tissues and deactivate virus. Total RNA and DNA were isolated following the manufacturer’s protocol and resuspended in 25 and 200 μL of RNAse-, DNAse- and proteinase free water (Sigma) for RNA and DNA, respectively.

### Quantitative real time PCR to detect PCV2

A quantitative real-time PCR (qPCR) was performed in the 8 tissues from all animals to quantify PCV2 load. Probe and primers used for the procedure were those designed and used in [22]. The mix for the qPCR contained 900 nM primers PCV2F and PCV2R, 150 μM probe, 0.4 μL IC kit, 12.5 μL Taqman Universal Master Mix and 2.5 μL template. Nanopure autoclaved water was added to final volume of 25 μL. The conditions for the amplification were 10 min at 95 °C, 2 min at 50 °C and 40 cycles of 15 s at 95 °C and 1 min at 60 °C. Triplicates of each sample were used for the qPCR.

### RNA integrity and quantification

Total RNA was quantified using ND 1000 Nanodrop® Spectrophotometer (Thermo Scientific, Wilmington, USA). RNA integrity and quality was analysed using the 2100 Expert Bioanalizer (Agilent Technologies, Santa Clara, USA). Total and low size RNA measurements were carried out. Total RNA was calculated using the Eukaryote total RNA Nano Series II software and specific chips (Agilent Technologies, Santa Clara, USA), while low size RNA was measured using the Small RNA Nano software and specific chips (Agilent Technologies, Santa Clara, USA).

### Small RNA library construction

All procedures were carried out using the miRCat™ microRNA Cloning Kit (IDT, Iowa, USA) protocol as basis, with the modifications described in [23]. For the enrichment of the small RNA fraction, slices were cut from a 12% denaturing (7 M Urea) polyacrylamide gel, using a miSPIKE™ (IDT, Iowa, USA), as internal size marker. Small RNA fractions were purified using Performa DTR gel filtration cartridges (EdgeBio, Gaithersburg, USA). A double ligation was carried out using 3′ and 5′ linkers from miRCatTM kit (IDT, Coralville, USA) in two steps. The 3′ preadenylated linker was coupled to the small RNA fraction in a reaction in which the mix contained 1X Reaction Buffer for T4 Ligase (Thermo Fisher Scientific, Walthman, MA) without ATP, 0.1 mg BSA (Fermentas), 1U T4 RNA ligase (Fermentas) and 2.5 μM 3′ linker. 6.5 μL small RNA fraction was added and incubated at 37 °C for 2 h. The 5′ linker was coupled in a mix with 1X ligation buffer (Ambion), 1U T4 RNA ligase (Ambion) and 50 μM 5′ linker in presence of ATP. 7.5 μL 3′linked RNA was added to a final volume of 10 μL. The mixture was incubated for 2 h at 37 °C.

### RT- PCR

RT was carried out with Super Script III Reverse Transcriptase (Life Technologies). Initial pre incubation at 65 °C for 5 min with 0.5 mM of each dNTP, 0.5 μM Primer mir5 and 11 μL RNA were carried out. In a second step a mix was prepared with 1X First-Strand Buffer, 5 mM DTT, 0.2 U RNaseOUT and 10 U SuperScript III RT in a final volume of 20 μL, the mix was incubated at 55 °C for 90 min, and 70 °C for 15 min.

PCR amplifications were performed using Expand High Fidelity Plus PCR System (Roche). Briefly, 0.05 U Expand High Fidelity Plus Enzyme Blend, 1X Expand High Fidelity Plus Reaction Buffer with 1.5 mM MgCl_2_, 0.2 μM Primers A5 and B3, 0.2 μM of each dNTP, 4 μL cDNA and RNAse Free Water to a final volume of 50 μL. Primers A3 and B5 were designed as fused primers with sequence primers mir5 or mir3 (IDT, Iowa, USA) plus sequence primers A or B (454 GS-FLX Roche), respectively, labelled with a five nucleotide sequence tag for differentiating libraries. PCR conditions were 94 °C for 3 min, 20-25 cycles at 94 °C for 30 s, 57 °C for 45 s and 72 °C for 1 min followed by 71 °C for 7 min. The number of cycles was optimized for each library to avoid saturation. The resulting PCR was purified with the QIAquick PCR purification kit (Qiagen).

Cloning in pGEM-T Easy Vector System II Kit (Promega) and posterior Sanger sequencing with the ABI-PRISM 3700 (Applied Biosystems) were carried out to check the libraries construction prior to the high throughput sequencing.

### High throughput sequencing

Amplicons were quantified using Qubit™ fluorometer, Quant-IT™ (Invitrogen™, Carlsbad, USA), prepared to a 10^11^ DNA molecules/μL and equimolecular pooled. Libraries were sequenced following the manufacturer’s protocol with the 454 GS-FLX System (Roche) at the DNA sequencing facilities at CRAG (Centre de Recerca Agrogenòmica, Universitat Autònoma de Barcelona, Spain). Sequencing data have been submitted to the European Nucleotide Archive (ENA) [24] with the accession number PRJEB7603.

### Sequence processing scheme

Primer sequences were trimmed and only those insert sequences between 15 and 29 nucleotides and with total number of sequences ≥3 were kept for further analysis.

For porcine miRNA profiling, sequences were compared to all available miRNA sequences (miRBase v20) using local Blast. Parameters were set to 100% identity and up to 4 mismatches allowed at the end of the sequences to solve the miRNA variability on 3′ and 5′ ends [25].

Differences in host miRNA expression were assessed. The total number of sequences obtained for each porcine miRNA was normalised by library size (in counts per thousand) and, then, averaged by group. Fold changes (FC) between groups were calculated using normalised data. Only miRNAs up or down-regulated for all individuals per group were taking into account.

### miRNAs target prediction and biological functions

For the in silico study of the potential targets, the DIANA-microT v5.0 web server with an adjusted threshold of 0.7 was used [26,27]. There were no porcine genes in the databases, so the study was done using the human genome assuming sequence conservation [28]. In order to identify potential targets in the viral genome, miRanda program was used with the following parameters: -sc 140 –en 20. Potential targets were then studied employing Web-based Gene Set Analysis Toolkit (WebGestal) [29,30]. The analysis was done using the Kyoto Encyclopedia of Genes and Genomes Database (KEGG) to check the biological pathways in which miRNAs are involved. The selected parameters for the study were the multiple test adjustment by Benjamini and Hochberg [31] and the significance level set at 0.05.

## Results

### Experimental infection

Until 21 dpi no animals developed clinical signs. PCV2 infection was confirmed in inoculated animals by qPCR in 6 out of 8 tissues: Spleen, inguinal lymph node, tonsil, MLN, lung and mesenteric lymph node, confirming the subclinical nature of the infection (Table 1). No amplification was observed in non-infected animals or in kidney and thymus of tested animals. PCV2 detection by in situ hybridization was negative for all samples tested. No histological lesions were observed at necropsy. MLN and tonsil were selected for small RNA library construction because of their viral load and because tonsil is the primary replication site.

**Table 1:** **Genome copies/mg detected by qPCR in tissues of infected (number 3 to 6) and non-infected (number 1 and 2) pigs with PCV2**

	**Animal number**					
**Tissue**	**1**	**2**	**3**	**4**	**5**	**6**
Spleen	-	-	3.2 × 10^2^	2.9 × 10^2^	7.2 × 10^1^	4 × 10^2^
Inguinal ln	-	-	1.2 × 10^4^	3.3 × 10^2^	1.7 × 10^3^	8.9 × 10^2^
Kidney	-	-	-	-	-	-
Tonsil	-	-	5.2 × 10^1^	1.8 × 10^3^	2.1 × 10^3^	8 × 10^2^
Thymus	-	-	-	-	-	-
Mediastinal ln	-	-	8.2 × 10^3^	1.9 × 10^4^	3.8 × 10^4^	4.6 × 10^4^
Lung	-	-	6 × 10^1^	2.1 × 10^2^	1.1 × 10^2^	9.2 × 10^1^
Mesenteric ln	-	-	2.5 × 10^2^	7.6 × 10^2^	7.2 × 10^1^	1.1 × 10^3^

### miRNA sequence annotation

A total of 12 small RNA libraries were constructed and sequenced. From the total reads obtained (1 106 437), after trimming the adaptors sequences, and selecting inserts ranging from 15 to 29 nt, a total of 796 710 reads were obtained (Table 2). Reads were aligned to the miRBase database (v20), not allowing any changes inside but allowing a maximum of four changes in the sequence extremes. Finally, 562 483 reads (4700 unique sequences) were aligned to miRBase. A total of 508 miRNAs were described.

**Table 2:** **High throughput sequencing reads**

Total reads	1 106 437
Trimed, not empty reads, ranging from 15 to 29 nt	796 710
Aligned to miRBase	562 483
Unique sequences	4700
Number of miRNAs	508

### Differential expression analysis

miRNAs were considered differentially expressed (DE) when fold change (FC) difference between infected and non-infected animals was higher than 5 and when the up- or down-regulation was conserved for all animals per group. From the 119 miRNAs highly expressed (>80 reads) in MLN, 8 miRNAs were DE, five up-regulated (mir-126-3p, mir-126-5p, let-7d-3p, mir-129a, mir-let-7b-3p) and three down-regulated (mir-193a-5p, mir-574-5p and mir-34a) (Table 3). In tonsil, no miRNA was DE from the 115 miRNAs with more than 80 reads.

**Table 3:** **Differentially expressed miRNAs in the MLN**

**miRNA**	**Inf/non inf**	**Reads**
ssc-miR-126-5p	6.28	18 794
ssc-miR-126-3p	7.51	17 050
ssc-miR-193a-5p	−54.29	3139
ssc-let-7d-3p	9.3	770
hsa-miR-574-5p	−19.9	310
ssc-miR-34a	−5.34	165
ssc-miR-129a	8.28	115
hsa-let-7b-3p	6.5	83

### Target prediction and functional analysis

Diana micro-T was employed to identify putative targets for eight selected DE miRNAs. A list of 5502 target genes was identified (see Additional file 1). A gene ontology (GO) enrichment analysis was used to identify the functions of the target genes (Table 4). No significant related pathways were found for mir-let-7d-3p and ssc-miR-126-3p. Some pathways related to viral infection process and immune response were found to be significant, such as the T cell receptor signalling pathway, Fc gamma R-mediated phagocytosis and the Fc epsilon RI signalling pathway. The prediction of putative targets in viral genome indicated that two of the DE miRNAs targeted the PCV2 genome: mir-let-7d-3p and mir-129a, both targeting the Cap gene.

**Table 4:** **Genome pathways predicted for selected miRNAs from Kyoto Encyclopedia of Genes and Genomes, related to the immune system and PCV2 pathogenesis**

**miRNA**	**Genome pathway**
ssc-miR-126-3p	-
ssc-miR-126-5p	T cell receptor signalling pathway
	Natural killer cell mediated cytotoxicity
	B cell receptor signalling pathway
	Fc epsilon RI signalling pathway
	Chemokine signalling pathway
	mTor signalling pathway*
	MAPK signalling pathway*
ssc-miR-193a-5p	Cytosolic DNA-sensing pathway
ssc-let-7d-3p	-
ssc-miR-34a	Fc gamma R-mediated phagocytosis
	Hematopoietic cell lineage
	B cell receptor signalling pathway
	Fc epsilon RI signalling pathway
	T cell receptor signalling pathway
	Natural killer cell mediated cytotoxicity
	Leukocyte transendothelial migration
	MAPK signalling pathway*
hsa-miR-574-5p	T cell receptor signalling pathway
	MAPK signalling pathway*
hsa-let-7b-3p	T cell receptor signalling pathway
	Chemokine signalling pathway
	Fc gamma R-mediated phagocytosis
	Leukocyte transendothelial migration
	NOD-like receptor signalling pathway
	Hematopoietic cell lineage
	B cell receptor signalling pathway
	Toll-like receptor signalling pathway
	MAPK signalling pathway*
ssc-miR-129a	Fc epsilon RI signalling pathway
	B cell receptor signalling pathway
	Chemokine signalling pathway
	MAPK signalling pathway*

*Signal transduction.

## Discussion

miRNAs can be an important factor playing a role in virus/host interaction and in the immune response involved in the pathogenesis of PCV2 infection, in both subclinical and clinical scenarios. This work is the first study on miRNAs gene expression in pigs infected with PCV2 using a deep sequencing approach. In the experimental infection carried out, the lack of clinical signs, histological lesions and viral detection by ISH and the low viral load detected by qPCR is in agreement with previous studies where a subclinical infection has been developed [32].

Taking into account the comparison of the most expressed miRNAs in both tissues (CN > 80), 74.8% of the miRNAs were common. This is in concordance with the lymphoid origin of both tissues. When comparing the tissues analysed, the expression profile was affected differentially by the PCV2 infection. The miRNA expression was affected notably in MLN, whereas in tonsil, the miRNA expression pattern was less affected. A possible factor affecting this expression is the difference in viral load. However, differences in cytokine expression have been described between tonsil and others lymph nodes [9] although the basis of this differential expression is not clear. Thus, the conserved miRNA expression in tonsil could be associated to this different cytokine expression. On the other hand, tonsil has been suggested as a primary replication site for PCV2 [2]. Because all animals were euthanized at day 21, the expression of miRNA could be altered by the differences in the period of time when the tonsil and MLN were infected [33].

Differential expression of miRNAs in MLN is high due to the PCV2 infection; from the most represented miRNAs in both tissues (CN > 80): mir-126-3p, mir-126-5p, mir-let-7d-3p, miR-129-2-3p and mir-let-7b-3p were up-regulated in MLN of PCV2 infected animals, while miR-193a-5p, miR-574-5p and miR-34a-5p were down-regulated.

miRNAs DE could regulate genes that would be involved in the immune response, such as T cell receptor signalling pathways, natural killer cell mediated cytotoxicity, the B cell receptor signalling pathway, the chemokine signalling pathway, Fc gamma R- mediated phagocytosis, leukocyte transendothelial migration, the cytosolic DNA sensing pathway, the NOD-like receptor signalling pathway, hematopoietic cell lineage and also related to Fc epsilon R-I signalling pathways.

miR-126-5p is the most represented miRNA DE in MLN and has been involved in the intracellular expression of Toll-like receptors (TLRs) 7 and 9 by plasmacytoid dendritic cells, producing (anti-viral) type I interferons [34], thus, mir-126-5p can modulate the physiopathology of the immune response against PCV2. The extracellular signal-regulated kinase (ERK) signalling pathway, one of the three mitogen-activated protein kinase (MAPK), has been shown to be involved in the induction of autophagy by PCV2 [35] and the inhibition of ERK signalling pathways increases PCV2 replication [36]. In our functional analysis, miR-126-5p was associated with the MAPK signalling pathway, therefore, this miRNA could be involved in both processes.

TFPI (tissue factor pathway inhibitor) is one of the genes targeted by miR-126-5p. Comparison with a previous study [32] in which gene expression in response to PCV2 infection was analyzed by microarray hybridization, showed a TFPI gene down-regulation in MLN. This decreased expression could inhibit migration and cell proliferation and thus, could be involved in the inflammatory process developed in pigs with PCV2-SD [32]. On the other hand, in a coxsackievirus infection, miR-126 targets SPRED1, LRP6, and WRCH1 genes, mediating cross-talk between the ERK1/2 and Wnt/β-catenin pathways, enhancing viral replication and contributing to the viral cytopathogenicity [37].

Target prediction analysis indicated that miR-126-3p was potentially able to regulate only 20 genes but no related pathways were found. Interestingly, one of the targeted genes is the previously mentioned SPRED1, shared with miR-126-5p.

miR-let-7b-3p was up-regulated in MLN and has been shown to be able to potentially regulate a great number of genes, thereby several immunological pathways have been found for this miRNA. Besides this, this miRNA has also been associated with the MAPK signaling pathway. Target prediction analysis showed that miR-let-7b-3p can regulate the expression of the AP1S3 gene, which is involved in the modulation of Toll-like receptor 3 (TLR 3) signaling [38].

mir-let-7d-3p was up-regulated in MLN, although 13 genes were found as potentially regulated by this miRNA, no related immunological pathways were found. miR-193a-5p was down-regulated in medistinal lymph node, and could potentially regulate genes related to the cytosolic DNA- sensing pathway.

miR-34a-5p is down-regulated in MLN and this regulatory role was predicted to be involved in several immunological pathways and the MAPK signalling pathway. In this case, target prediction analysis revealed that miR-34a-5p targets KLRK1, killer cell lectin-like receptor subfamily K, member 1, which binds to a diverse family of ligands that include MHC class I chain-related A and B proteins and UL-16 binding proteins, where ligand-receptor interactions can result in the activation of NK and T cells [39]. It also regulates ARHGP6, a member of the Rho family that participates in endocytosis processes. PCV2 internalization is produced mainly by endocytosis, mainly through Rho- GTPase mediated dynamin- independent pathway [32].

miR-574-5p was down-regulated in MLN and was predicted to be involved in the regulation of the T cell receptor signalling and MAPK signalling pathways. miR-129-2-3p was down-regulated in MLN, predicted pathways in which this miRNA was involved were the Fc epsilon RI signalling, the MAPK signalling, the B cell receptor signalling, and chemokine signalling pathways. This miRNA has previously been described as targeting transcription factor SOX4 which has a critical function in normal B-cell ontogenesis [40].

Some of the predictions described in this work could be involved in the manifestation of the pathogenesis of the subclinically infected animals, but at this point, we want to indicate that further functional assays are needed to confirm the computational predictions of miRNAs target genes and the regulation of the pathways described.

The effect of host miRNAs on viral gene expression was analyzed using miRanda. Thus, mir-126-3p and mir-126-5p, the most represented DE miRNAs, showed no targets in the viral genome, while mir-let-7d-3p and mir-129a presented targets in the Cap gene of PCV2. Both miRNAs are up-regulated in MLN and can affect viral replication as has been seen for others viruses where several miRNAs can down-regulate the expression of viral proteins [41,42]. Nevertheless, the role of host miRNAs in the regulation of the viral replication is controversial as has been revealed in an extensive study [43]. The authors showed that many human viruses are refractory to inhibition by host miRNAs due to miRNA driven viral evolution. Thus, experimental assays are needed to clarify if PCV2 is regulated or not by porcine miRNAs.

The identification of the DE miRNAs and the prediction analysis regarding the functions of these miRNAs in an in vivo PCV2 infection in its natural host has been described in this study. The two tissues analysed presented a different behaviour in their miRNA expression pattern in response to the infection.

## Additional file

Additional file 1:
**In silico target genes predicted for the eight selected DE porcine miRNAs in the infected and non-infected pigs.** List of target genes predicted for the eight selected DE porcine miRNAs in animals infected and non-infected with PCV2, isolate Sp-10-7-54-13. Only ssc-miR-126-3p and ssc-let-7d-3p presented a low number of target genes (≤20).
