# Supplementary material for: Identification of microRNAs in PCV2 subclinically infected pigs by high throughput sequencing
Source: Vet Res. 2015 Mar 3;46:18. doi: 10.1186/s13567-014-0141-4 (PMC4346106; doi:10.1186/s13567-014-0141-4)
Supplement: Additional file 1: — In silico target genes predicted for the eight selected DE porcine miRNAs in the infected and non-infected pigs. List of target genes predicted for the eight selected DE porcine miRNAs in animals infected and non-infected with PCV2, isolate Sp-10-7-54-13. Only ssc-miR-126-3p and ssc-let-7d-3p presented a low number of target genes (≤20). [file 13567_2014_141_MOESM1_ESM.docx]

| **miRNA** | **Predicted target genes** |
| --- | --- |
| ssc-miR-126-3p | PTPN9, PLXNB2, CTD-162K18.4, KANK2, ITGA6, ZNF556, ZNF219, SPRED1, PLK2, IRS1, SLC37A3, PMM1, CAMSAP1, FBXO33, C20orf26, DIP2C, TNFRSF10B, EFHD2, SLC7A5, ZNF131. |
| ssc-miR-126-5p | ESRRG, RFX4, MDM4, SKOR1, HOXA13, A2ML1, HSPB8, AL353698.1, EGFR, MAP3K2, INPP5D, GRIK2, TRPS1, TRIM8, S1PR3, ARL11, FAM168A, SUGP1, GNE, REV3L, CASK, DENND1B, ZBTB7B, FGF7, NCR3LG1, PREX2, NAT1, MFAP4, UBN2, GABRB2, CPED1, MACC1, BAI3, BZW1, SOX5, SH3BGRL2, FXN, NAF1, ZNF738, EGFL6, NFYA, AP3B1, TSC22D4, KIAA1456, ZNF33A, PLCB1, GABRA4, HOXC12, ANO5, CALHM1, KIAA1644, C11orf87, SLC25A53, SULT6B1, L2HGDH, FAM9C, TNFAIP8L3, ING3, TMEM56, TBC1D3C, PDZD8, PAX2, GIN1, WFDC13, RABL3, TBCA, SLFN5, TMEM41B, FAF1, EIF3J, PPP4R4, CHI3L2, YIPF4, BRWD3, LAMTOR3, CAMK2A, GGT6, PTPN20B, HOXC8, RAB30, KL, NDUFS1, EIF2AK2, CSRNP3, JARID2, RASAL2, ROCK1, PTPN20A, JPH1, PTPN12, PLEKHG7, AMFR, PDE7B, COLEC12, MBOAT1, CLEC1A, FBXO6, GCLM, MGAT3, TMEM182, MMRN1, RP11-849H4.2, CD44, LYPLA1, MAN1A1, SEPT7, CHD1L, FLT4, STPG2, SCD, ZNF687, CHMP5, TRDN, OSTN, MIER3, MAK, KIAA2018, CCDC88A, ZNF33B, MR1, FLRT2, PDLIM5, IL23R, CHM, INO80D, FOXN3, ZNF627, HOXD3, CNOT4, EIF2A, CCDC13, AGAP7, NPAS2, GULP1, TUBGCP4, CSNK1G3, DDX59, NCOA7, TMEM33, GRID1, LRRC55, TTF2, EYA1, PPIL1, HIPK2, MLL, SERF1A, ADAMTS6, AC025278.1, GJC1, SOX6, CD84, ZNF519, NCOA2, COMMD2, IDS, DICER1, AKAP6, PNRC1, STXBP4, RBM26, MAT2B, ZDHHC15, STC1, STEAP2, SKIDA1, PIK3CA, HOMEZ, C8orf49, CCL28, CREB1, C12orf55, COMMD10, FAM27E3, QRSL1, ERGIC2, FZD3, CDCA7, RASEF, FPGT, TMPO, TMEM237, GPA33, C12orf50, CDK13, DNM1L, EREG, CYS1, GPR85, RORA, BICD2, SLC6A15, PPARGC1A, ZNF354C, GPR88, ST8SIA3, RSBN1, TBC1D15, LPP, LRCH2, LTN1, C4orf29, MSRB3, EBF1, TMOD2, HOXB6, HELZ, C18orf25, DTL, RGS18, FAM111A, PTPRD, CA1, AC130352.1, PTPN4, TRPC5, RGR, FBN2, HSD11B1, ZNF207, KRT78, ETV1, SKAP2, CADM2, CSF1, USP12, KLHL32, RP11-17A1.2, SPATA2, WDR35, AP1G1, NSUN3, DHX33, SPAST, MAPK10, OTUD4, EFCAB11, ZNF503-AS2, RAD50, ANKRD32, SLC2A13, N4BP2, ADCYAP1, FAM102B, LPPR5, CPEB4, DIRC2, EPHA7, GBP1, UBE2W, GNAO1, KCNT2, ARL13B, SAMSN1, ZBTB7C, FAM199X, LRRC42, RP11-671M22.1, VIL1, NUDT12, NWD1, MTL5, RGS5, MANEA, ARSK, SPATA6, RUNX1T1, ZFP30, F9, ZNF10, RAB8B, FBXL8, KIAA1468, HPSE, C2CD4A, TMEM26, HECTD2, DOK6, PI15, CMC1, PAX9, THAP3, RP11-463J10.2, MON2, ZC3H6, EXPH5, TCHH, LANCL3, CLCN3, EFHA2, CREB5, TMEM65, NHLRC2, ZC3H12B, ISG20, ACTR1B, KCTD3, C10orf126, FSD1L, C12orf39, SGPP1, C2orf69, DAB2IP, PDGFD, RP11-392O18.1, ADAMTS4, TNRC6A, SMAD4, CACNB4, CLEC12B, RNF19A, LUC7L2, EVX2, PLEKHH2, ASAH2C, FAM27E2, GRIA2, ZBTB8B, CALHM3, C8orf37, AZIN1, PRRG2, MS4A1, FAM216B, PAX6, BCL11B, AP000708.1, PGM3, ZC3H8, CBFB, BLZF1, CDH8, SYNPO2, SPATA18, FAM27E1, CNTN1, CELF2, ERO1L, DRGX, CTDSPL2, PRTG, CD200R1, MCHR2, SREK1IP1, EFR3A, EYS, PTGS2, ZPLD1, ELK4, TNKS2, C9orf41, PDGFRA, ERAP1, COBLL1, SRD5A3, RBM46, FBXO3, LDB2, IL7, ZFY, PRRG3, ATXN7, ASAH2B, NFAT5, AIMP1, MMS22L, TSHZ3, LOX, TMX3, RP11-67H2.1, MRPL42, IFFO1, STK17B, GPR12, XRN1, ZC3H7B, DMRT2, DDR1, SLC35A5, MBOAT2, NDUFA9, SPICE1, GPBP1, BRAF, SOCS6, VSTM2B, ABCA5, ASAH2, CAMK4, SRFBP1, IL15, LONRF2, GLIPR1, ZIC4, ZNF695, MKLN1, RNF175, ZFHX4, SSX2IP, RALGPS2, RIMS1, REEP3, PAQR3, CORO2A, POLR2K, GXYLT1, BRD3, ATF7IP2, PRRC2C, CEPT1, BACH1, E2F7, SGCB, HMGA2, METTL2A, FAM165A, FAM183B, LHX9, MYEF2, C21orf62, MBLAC2, RBM12B, CD2AP, FRYL, DACH1, TMEM2, SSBP2, RTKN2, RFTN2, TAOK3, CHD9, CYB561D1, UBXN2A, SLC30A1, OCLN, ZNF703, ZNF148, HMGXB4, TET1, SERF1B, AQR, FAM78A, ARID2, DST, FBXL14, PLA2G12A, PPP1R1C, DMXL1, RPS6KA3, AHCTF1, BNC2, CPEB2, PLAG1, KPNA5, POU2F1, CPNE8, C9orf114, ATXN7L1, SYS1-DBNDD2, AGTR2, PELI2, ZNF583, GOLGA8F, REXO1L1, MCTP1, FZD6, MIB1, ZC3H13, GJB2, TFEC, ITGB8, MAP9, ZNF777, PHLPP2, SCRN1, PCSK2, SEMA7A, C14orf118, HOXB2, DYNLT3, C6orf89, PPP1R10, BICC1, MLPH, RAPGEF6, CLASP1, TFPI2, LCP2, FPGT-TNNI3K, ITFG1, HOXB5, OXSR1, CCDC111, IGJ, GABRA5, STYX, PDE10A, DUSP28, AGPS, PLOD2, SLC25A30, PTAR1, SLIT2, KLF17, EDA2R, KCND2, GPR110, PYGO1, GOLGB1, C12orf40, C5orf47, NF1, UGT2A3, SAMD4A, GTF2A1, SH2D1A, SC5DL, CEP97, TNFSF4, NR2F1, ARHGAP20, PPP1R15B, PDE3A, CHRNA5, FAM13B, FAM135A, VAPA, DCLK3, CDK6, ZNF254, GNAQ, SBSPON, KIAA0355, ICK, ELAVL3, USP47, RP11-108K14.4, FAM76B, ERBB2IP, ZIC1, TCEA1, DIMT1, TNFAIP8, ITGA8, BTC, SEC62, CPD, MOB1B, LHCGR, SLC5A3, PCGF5, ROCK2, SRSF12, GUCY1A2, PRKG1, DCUN1D3, NPAS3, FOXP2, CCNT2, CHST7, SEMA3D, ZNF566, TRHDE, NEUROD6, ZNF720, C5orf51, SLC17A8, UAP1, SORD, U2SURP, PTBP3, DYRK2, MPP7, C12orf23, ABCB5, PAX5, PANK3, ENPP5, ZCCHC5, LRRC3B, CCDC149, MOG, HELB, DAPP1, VTI1A, ZNF536, PURB, SEC61A1, GPM6B, BMPR1B, ZDHHC21, ZNF681, AXIN1, FAM198B, ZNF383, G3BP1, IL36G, TRAF6, RBBP9, SNX18, ZFP14, APOPT1, PDZD2, C1QTNF7, LIMCH1, JRKL, RP11-169F17.1, C11orf45, BAG3, EPHB1, GLRA3, VWC2L, POFUT1, SLITRK4, BTF3L4, MYOZ3, HCN4, CCPG1, PTGFR, HRH4, FAM151B, SPRED1, MTCH2, RP2, HTR1F, SLC35A3, EYA4, NPR3, KRAS, GMNC, NEGR1, PLEKHB2, TFPI, MAP3K7, ABCD3, FMO2, FAT3, ADO, PIKFYVE, SMARCC1, NLE1, GSPT1, INSC, FAM63B, PPP1R12B, PAPPA, SOS1, BTAF1, ZNF81, DLK1, ZFR, ARL5A, KIRREL, MED14, TNFAIP2, QPRT, TCFL5, GIMAP8, FAM129A, HMGB1, TMEM248, NR1D2, PFN2, SLC2A11, GMPPB, PNLIPRP3, SLC35G1, SYT5, POU5F1B, APPBP2, RUNX2, RFX3, CBWD3, ANGPTL7, DCUN1D1, SPRY3, POC1B-GALNT4, NDUFV2, TCF12, NEUROG2, VWA8, OAS1, ELAVL4, UBTD2, ATF7IP, MSR1, GAB1, C1GALT1, GOSR1, RBM19, C1orf141, SRRM1, ANKIB1, CCR1, CEP78, LRRC8B, ZDHHC17, NRK, RCOR3, IKZF2, ENOX2, PDE4DIP, PTEN, C21orf91, MARCKS, C12orf65, KLF12, IMPAD1, CBLB, HGF, HEATR5B, CYP4F3, HLF, VTA1, ABCE1, ASXL2, USP10, IGSF1, CDC14B, SEPSECS, ADK, TMEFF2, BTBD3, GPR27, GABRA2, SPAG9, MTO1, SOD2, SLC26A4, MITF, TDRD3, PAWR, C19orf69, ANKRD34B, LMO7, ENPEP, MCTP2, FAM189A1, PRUNE2, RP11-59H1.3, EZH2, SLC12A2, MLLT10, OSBPL8, SLC41A2, ARMCX3, C5orf44, MDGA2, C7orf57, HIBCH, ZMYM6, TOB1, ARHGAP28, HDAC2, BTLA, ANTXR2, PAPD5, PRKX, NETO2, C4orf46, RAPGEF2, SREK1, FMN2, PROCR, ENPP6, FAM221A, CUL2, UBXN7, RASA2, TMEM135, FAM179B, RINL, SRSF10, ERMN, SETBP1, GRB2, PEX5L, PHTF2, NIPA2, ATL3, RCOR1, ASPN, JMJD1C, TSHR, ZDBF2, SHISA3, TWF1, PIP4K2A, TRDMT1, GABRA1, ZBTB38, MOB3B, STARD4, SGTB, CBWD6, TENC1, CCP110, LMBRD2, KCNJ16, ARHGAP18, CA3, ABRA, ZNF280D, GATA3, SLC7A2, TTC39C, RP11-73M18.2, NBEAL1, KIRREL2, PNPLA8, HDAC4, SH2D4B, ADAM22, BEND3, CASP3, BCAT1, MYO3B, KCNJ3, ABCC9, SPAG17, STRN3, SYT14, TTC7B, ALCAM, KIAA1033, VCPIP1, HLTF, RNF217, STAM2, EPGN, ERVFRD-1, KIF15, ZBTB1, MED17, CCDC38, FAS, ZNF624, CHRNA7, RBM41, SLC38A6, LACC1, NTRK3, FGGY, ATG10, MT1H, C11orf82, AP1S3, ZNF295, ZNF674, ABCA1, PIK3C3, ATXN1L, TRIM64B, GABPB1, FAM8A1, PALLD, HBS1L, USP15, P2RY12, GCC2, MBD5, CHP2, PDS5B, DCX, C10orf53, HACE1, VPS13C, CRISPLD1, SNCA, SLC30A6, USP14, SLC26A2, WDR48, AC008132.1, C5orf24, SYS1, REG3G, ONECUT2, KLHDC10, NREP, SLC10A7, CHPT1, LATS1, CLLU1, SPACA1, MID1, CSMD1, SLC16A7, MYBL1, TMEM39A, RAD21-AS1, VGLL1, NEUROD4, NCALD, KCNV1, C8orf34, MAPK1IP1L, PHLPP1, SCAP, TMED7, ZBTB26, AKD1, USP46, ATP6V1G2, METTL6, TBR1, GOLGA8G, MAFB, PLCXD3, GLS, CUBN, TRMT10A, BMP3, PCDHA6, RB1CC1, RP11-766F14.2, CPA3, E2F6, PRDM8, TOX, SEC11C, SHROOM3, ZNF546, ATF6, NDFIP1, NUAK1, UHRF1BP1L, TCHHL1, KCNJ2, DSG4, DLGAP1, RP6-24A23.6, WEE1, C15orf41, SLC6A5, MMP16, KIFAP3, GTF2F1, C8orf4, LONRF3, TET2, DGKB, DNAH5, SMC5, RAD21, LRRTM3, KBTBD6, PRKCA, FAM3C, PAK2, CNTNAP3, ADAM9, RAB9A, ZNF385D, GNGT1, ZBTB2, NFIA, OPA1, IL17A, NCAPG2, PRKAA2, C18orf32, PMS1, TMED2, DKK2, FYB, ATXN3, CALD1, BICD1, UACA, AL137003.1, ZNF347, C10orf129, NCAM2, NAA50, SFRP4, ANGPTL3, MTDH, EFCAB7, PTPRB, NLGN4X, DHFR, MRS2, CHCHD3, NR3C1, HIPK3, SOX13, CA12, CLOCK, PPM1B, TBC1D20, C14orf129, CCDC68, TMTC1, TMEM27, CHL1, CHRM2, KIAA1024, LINGO1, ZZZ3, ISPD, UBR1, CTAGE5, GLE1, ANGPT1, PEG10, CLIC2, DVL3, XIRP2, CRY1, SNX10, KBTBD7, KCNG3, SDE2, USP9X, ASPHD2, FAM201A, ITM2B, OTOGL, GCNT3, ZNF793, AL359878.1, PRAMEF13, MBD2, ITSN2, ADAMDEC1, LMLN, RALGAPA1, CENPJ, SESTD1, EXOC5, PLP2, GDF6, AMACR, HHIP, ZNF492, SNX15, SLC24A4, ATMIN, PLSCR4, BBS12, PGM2, ZNF385B, LRRC57, C5orf30, BPI, PNN, CXorf1, SLC25A37, C22orf45, DNAJB4, DLAT, HECW2, PTER, PGC, C4orf26, REEP5, IGF1R, SLC26A7, AC013461.1, TGFBR1, TGDS, RBBP6, UBE2H, ZFPM2, DLL4, ZNF273, NUPL1,, SPATS2L, WDHD1, CCDC53, FAM160B1, GOLGA8IP, FGD4, EIF3H, FAM190B, AKNA, SLC35F5, HNRPLL, CCDC50, TMEM123, CHAMP1, NABP1, DMBX1, KPNA4, ZNF567, MBL2, ASPH, NACC2, B3GNT5, NSUN7, TMED5, GINS1, PHYHIPL, CTD-2054N24.2, COL11A1, CTAGE9, FBXO36, FRZB, TEX15, NUS1, SYT10, KATNAL1, POLK, FOXR2, SSPN, STBD1, STRN, ZNF615, CASP9, CRB1, TLK1, EP400NL, TBC1D19, IRX3, EIF4A2, SLC25A24, JKAMP, LRP6, ANXA7, TCF24, CDH19, FNTA, PPM1A, EVI2A, C1RL, NCKAP5, LCLAT1, FTSJD1, FAM84A, C1orf27, ARHGAP24, UTY, CEP170, LYRM5, SLC38A4, GALNT4, YTHDC1, PIGK, MAP4, SGIP1, TBCK, GOLIM4, PATE1, PPP6R3, MTAP, EARS2, PSMA5, PARP12, SEMA6D, SASS6, KIF3A, CLEC1B, SPDYE3, AC005754.1, VMA21, ZNF333, CTCFL, TRNAU1AP, CTBS, PPIL6, COL12A1, HMGCS1, PANX1, MRP63, SLC25A40, TRIM33, IGSF10, HIF1A, RSRC2, CMYA5, AP1S2, FAM196A, RAB11FIP2, AK4, ARPC3, BCL2L2, ACYP2, SECISBP2L, PUS7, TRAM1, SESN3, SLC7A4, TMEM50A, VGLL3, FGL2, DCDC2, SLC1A1, PCDH18, KIAA1671, ATR, ST6GALNAC5, CCNYL1, MYSM1, KAT2B, THEMIS, CXXC4, IMPG1, ITCH, CCDC141, MBNL3, TOMM34, CRLS1, NOVA1, ARL4C, SORBS2, TMEM70, EBAG9, GRIN2A, HNMT, GAS2L3, HOMER1, RGS4, RAB9B, PRAMEF14, NFIB, ZBTB10, C15orf27, ADAM8, DUXA, FGFRL1, BMP5, KCNQ5, SIX4, SLC16A9, RP5-862P8.2, FRG2C, FNIP1, OTUD3, ANTXR1, PARP8, UNC79, PMP2, UTP14C, KIAA1383, SCN3A, SLC30A4, NANP, CLDN12, TRAF3, HIST1H2BF, AC093510.2, BPY2, LGALSL, MS4A3, BCAP29, HSPA4L, MEF2D, LRRK2, PPP4R2, AHR, SHH, IL1RAP, ZNF208, ROBO1, PIK3C2A, C7orf63, AKR1B10, RRP15, GFPT1, VPS13A, LRRC9, NCOA6, MED13L, KCNB2, PMPCB, AKAP4, PKIA, EBPL, NTF4, INPP4B, KERA, FAM122B, PEX3, C6orf118, SLC22A24, HS3ST3B1, ZNF100, FBN1, ERLIN1, PARP11, RNF41, TMTC3, LRRC8D, KRR1, RHOBTB3, RXFP1, KIAA1549, PRR23C, CCDC59, UBE2G1, BAIAP2L1, CSF2RA, PRDM1, ZXDA, BEND4, RAB3IP, HECA, NXT1, OR51E2, PM20D2, DCP2, MCM3AP, EPHA5, CDK19, NAA30, SUMF1, ADAMTS5, ELF5, YES1, RAB2A, FGG, RCBTB1, GPR180, MIER1, C14orf23, GLUD1, ABCA10, GRAMD1B, BHLHE41, EPT1, ZNF461, SLC1A2, MAP7, GRM1, CCDC132, RAB3GAP2, DCLK2, ZADH2, RALGPS1, ZIC2, DLG1, GABRP, CUL3, GSK3B, DDX10, RPRD1A, GREM1, OSBPL11, UPP2, ULK2, NOG, SPO11, ST13, GPR137B, PPAT, EPHA3, ZFX, ZNF23, FABP4, SPOPL, NHSL1, GALNT5, MGP, NCAN, FSHR, HP1BP3, ANGEL2, BCLAF1, XIAP, HINT3, GPR64, USP45, CRCP, GPR151, SOCS3, TOR1AIP2, AP000783.1, AMMECR1L, DCAF4L1, SNX13, GBP3, SLMAP, B4GALT4, HPS5, ALDH6A1, SPIN4, SOCS4, ATG12, IGF1, FBRSL1, LCORL, GPBP1L1, FAM120A, C4orf34, MAGEE2, MEGF10, RP11-1220K2.2, FGFR2, C15orf29, GRIA4, UGT8, CLDN22, SDPR, WBP2NL, GNRHR, CNTN4, HEATR1, ITGB6, IREB2, CHMP3, EMCN, TOP1, ITGB1BP1, ARMCX2, USP51, RIMBP2, TERF2, RP11-152F13.5, NFXL1, AC124890.1, CRK, WDR17, ARNT, CREBBP, UBE3D, PDE1C, CHEK1, DCK, TMEM183A, SPDYE1, UPRT, PPP1CB, PHKA1, GBP7, SYAP1, BARD1, B2M, UGT2B17, UBE2V2, SP1, ATG3, RABGEF1, MUC22, BNIP2, FAM82B, CDH9, LINC00493, FAM172A, YWHAZ, TMEM236, CDK12, ZNF800, FURIN, STK3, BCAR3, DSC2, TROVE2, TDG, CNPY1, REPS2, TCP10L2, ZBTB6, HNRNPA1, DPPA4, MSANTD4, CASR, PRKACB, ZWILCH, LIMS1, PIGX, SLC4A7, CANX, TSC22D2, FIGN, C15orf61, C14orf37, EEA1, MAGEB5, ATAD2, EGLN1, TTLL7, ASB4, ZRANB2, YAF2, GLRA2, PLA2R1, FAM18B2, KLHL24, FAM23B, ZNF697, ULBP1, COL4A4, XKR6, PRAMEF1, TTC37, LDLRAD2, PHF23, NEUROD1, IYD, ATXN3L, PCNX, RAG1, HERC4, BTN3A3, C15orf53, TOX3, RARRES1, TMEM55A, ZNF776, RASSF6, ZNF770, KLHL4, SNCAIP, AP1AR, GMCL1, ZNF26, COX5B, SDC2, PTPLAD2, OSTF1, CHRAC1, FNDC3B, IL1F10, MXRA5, ZNF253, KDM6A, MYT1, OSMR, STOX2, ASTN1, PCDHB13, CRTAM, THSD7B, LUZP2, FAM46A, AL953854.2, HPGD, PSG1, C14orf64, YAP1, SNRPF, ERI1, TMCC1. |
| ssc-miR-193a-5p | ZNF142, DMRT2, AC099552.4, ITSN2, USO1, ACVR1, UBE2D2, SIVA1, SFTA3, WIZ, CD200, ZNF141, RGS22, ERVFRD-1, VPS13B, C11orf72, SLC30A5, KDELR3, GANAB, HDAC2, BLID, PDE1C, UGGT2, CREB3L3, CEP120, RP11-697E2.6, SYNPO2L, AIF1L, OGFRL1, GPX8, THUMPD1, C22orf46, IPO8, PCDHA6, ALMS1, MLLT10, TRAPPC11, CCT3, RP11-152F13.5, CUL3, FADS1, MMP16, HSPB6, AGBL3, ZMYM4, COMMD6, PCDH10, OLIG3, NSFL1C, BLM, KLRD1, TSC22D2, RAP2A, KLK4, CALML4, C15orf32, ANGEL1, NT5DC1, BTG1, CALCRL, IQUB, TCF20, ZNF827, TMPO, EBF3, RP11-276H1.3, SMARCC2, ARMC1, DCLK1, CNTFR, ESCO1, NLN, NETO2, RARB, ZNHIT6, PTCHD1, CHMP1B, CRY1, ZNF628, DSCAML1, IK, ACSF2, C14orf118, XK, SRR, IQCF6, C1S, EXOC3L2, XYLB, AL137145.2, HEATR6, KCNN2, OTOR, ARHGAP6, NT5C1B, CDADC1, SPOCK1, GNAQ, APOC2, EIF2C1, PHACTR3, CEBPG, LINC00636, NDUFA9, ALG10B, RNF217, PEX11A, HABP2, VEGFB, RGPD1, TAX1BP1, LYST, GTDC1, AC008394.1, CLIP3, DDX31, BTBD10, LRRC6, BCL11B, EEF1A1, CNOT10, CMPK1, ASCC2, CHD1, MGAT4A, LAIR1, UBN2, GORASP1, CSPP1, CCDC38, EIF2S2, DSE, NOP2, AL023807.2, POTEC, KRT79, PROX1, TNS1, PXK, AC009113.1, SLC25A47, HDX, CSNK2A1, TRIM23, RHOBTB1, UBAC2, NBPF20, NOP56, GOLGA8A, VNN1, CLCA2, TSPAN2, LDB3, SF3A3, PPP2R5D, SIM2, ZNF238, CCDC150, IFNGR2, PHACTR4, KSR2, PYGL, AC009237.1, KIF18A, SAMD7, CREB3L2, ZNF434, SLC6A17, SNX17, RAB3IP, USP51, ABCD1, AADACL2, FDFT1, ZFP3, C14orf28, ARPP21, ELP5, ZNF669, WASH4P, ZFP90, LHX1, TTC26, CYP19A1, HTN3, ITK, GZMB, ARID1A, CYTH3, ZNF572, GPATCH8, HCRTR1, POLR3H, TMPRSS4, FPR3, NUFIP2, HSPG2, TMEM245, C2orf48, ERBB2, SBF2, NOVA1, MKL2, GOLGA8F, EZH2, ATP8B3, MLL, GBA2, FAM98B, IL12B, FAM83A, OLA1, AL391421.1, PIP4K2A, CYTH2, GOT2, CA7, RSPO4, OTUD7A, C15orf43, ZNF776, TRPC5, PAPPA, RGSL1, NTRK2, SCYL2, BMF, CPA6, GIPC3, CAMK2G, FBXO47, SKAP1, FRRS1L, RPRM, CHCHD5, MTMR14, FCAMR, KIAA1715, GOLGA6A, POU2F1, TIPARP, PPP1R16B, DNAH3, UBIAD1, SYT11, KLHL22, NKIRAS1, TCHH, FRMPD4, SLC25A39, ZNF687, CLK3, SPANXN5, DDX58, LVRN, FAM64A, CDC23, C8orf33, HUNK, TCN2, IFNA8, ATR, SHARPIN, ZNF57, MTPAP, TIMMDC1, C19orf57, ZSCAN20, KIAA1671, PCDHA1, BARX2, DENND1C, DSCR4, PPARA, C3orf18, KLK12, PPIL2, OVCH1, CBLB, C22orf29, DCN, PCDHA6, PSG4, DUSP27, DPH1, CD27, GRAMD3, ZHX3,, RSAD1, HOXA1, PCDHA4, PCDHA6, PCDHA5,, PCDHA6, HYOU1, XIRP1, SETD2, PCDHA2, PCDHA3, PCDHA8, PCDHA11, RBCK1, NET1, PCDHA6, PCDHA7, HDGF, KRIT1, TCEB2, GOLGA1, KIAA2022, BCL2L15, JAKMIP3, BICD2, UNC5A, DOLPP1, INMT-FAM188B, SPATA3, ANGPTL3, LTA4H, MT1G, RP11-1102P16.1, MYLK, HDHD2, DCAF13, SAPCD2, ROPN1, PLEKHG4B, PIP4K2B, WNK4, FAM98A, SLC24A6, GREB1L, NR1D2, PLEKHA2, FBXL3, SOGA1, MOCS1, ADAM22, C17orf66, MLL2, SIGLEC5, MUSK, MLL3, GOLGA8G, IL1RAPL1, KIF14, PAPOLG, DTX3L, DMKN, CD34, GMIP, KCND3, UBL3, IPPK, CNTNAP1, THEMIS, LTB4R, AR, NCAPD3, MPHOSPH9, MAGEB2, ZNF384, MTMR4, ARID5B, GOLGA8B, KRBA1, HSFX2, SLC18A1, FSTL1, SULF1, NCALD, ZNF774, S100PBP, CYGB, MBP, IAH1, GRM4, CNTN2, FXYD5, SLC7A1, HIST1H4E, WBP2, DCP2, DDA1, GPRASP1, SUV39H2, EXOC8, ZNF385C, RPS2, EIF2AK4, NCAPD2, TSEN15, TUBA1A, ASXL3, ENTPD5, YTHDF1, TLR3, SETD3, TGFB2, DBF4, MON1B, ST8SIA2, ZNF394, CELF5, CASP1, ZNF317, QARS, MAP4K1, ZC3H6, MAGI3, WDR74, SERPINA9, KLF7, CLEC1A, RGS11, CD247, C9orf89, CHST14, SAFB2, SCARB2, PCNXL2, CRADD, DST, IGF2BP3, ATP8A1, PRKAA2, NUP210, SNED1, OLFML2A, ZNF746, ADGB, LINC00675, NXF1, DNTTIP2, LZTS1, COL27A1, RPAIN, SRRM4, ATCAY, TEP1, WT1, SIRPA, RSF1, AL033381.1, RGP1, KLF17, CPEB2, CYP4F22, CYP46A1, FBXO17, STRC, PTPRK, NCAM2, MEF2B, OVCA2, RASSF3, BAAT, OVCA2, ITGA8, NRXN1, GRIK3, ANKS1A, PPP1R1B, GPR17, PRICKLE2, TNFAIP8, ITSN1, UGT1A4, SNW1, C14orf177, SHROOM2, CSF2RA, FBXO7, SRF, ILK, WARS, BUB1B, SPRY4, BSDC1, YES1, FXYD3, AL136419.1, SIGLEC6, ONECUT2, ZNF710, ANO10, SIGMAR1, LRPPRC, SPIN1, CEP78, PVR, HIBCH, AAK1, TNPO1, BDH1, RFX5, NBPF24, C1orf213, IGF2BP1, ZFAND1, SLC51A, FOXP4, LIX1, ZNF81, NCR3LG1, OMD, TRAT1, CNNM1, CHTF8, EMR1, CECR2, TMEM86A, DNAH1. |
| ssc-let-7d-3p | AKAP6, ZIC1, SIGLEC6, KCND3, PTAR1, CDH19, AUTS2, NOM1, WASL, MOB4, DGKI, YY1. |
| ssc-miR-34a | GABRA3, E2F3, HCN3, CCNE2, RRAS, TRIM67, VAMP2, ACSL4, GOLPH3L, SATB2, TGIF2, DBC1, ZNF281, FAM70A, SYT1, PPP1R11, FUT8, NAV3, FAM76A, SIRT1, RP11-368I7.4, C20orf24, PPFIA1, LEF1, SLC25A27, NRIP3, MYCN, OSGIN2, NUMBL, E2F5, DCUN1D4, MAP2K1, BRPF3, ZDHHC17, TBL1XR1, PPARGC1B, MGAT4A, FAM123A, TPD52, FAM27D1, PPP2R3A, PLAG1, EIF2C4, PSMB6, SENP1, MLLT3, SGPP1, HNRNPA1, SEH1L, FGD6, RGS17, PNOC, STRN3, MSL2, C21orf7, ZDHHC16, ANAPC4, TMUB2, C6orf120, CBFA2T3, ZNF644, OLIG3, TMED8, AL049840.1, VAT1, GPR158, PVRL1, C14orf43, ACSL1, MOAP1, ATXN7, FAM135B, INA, ESYT3, CR2, ZMYM4, BMP3, ZNF142, GALNT7, AREGB, SPICE1, PACS1, SERPINF2, UBP1, CAPN6, FOXJ2, DCX, NOTCH1, NAV1, FOXN2, ELMOD1, CACNA1E, SGTA, DGKZ, DDX17, EVI5L, PCBP4, ASIC2, SWT1, KLRK1, SOGA1, PDGFRA, KLF4, CERS6, BCL2, DLL1, PURB, LGR4, VAC14, KCNK3, ZNF551, XYLT1, ADK, C14orf28, MTNR1B, JMJD1C, SNX15, SCN2B, GINS3, FAM123B, GAS1, NSD1, DACT2, DAAM1, LRRC40, BTBD11, TBCK, HOXA13, PPP1R16B, POGZ, PROX1, ZFHX4, PTPN4, TNRC6B, USP54, FAM167A, AREG, FLOT2, ARHGAP36, PRKCE, DNAH14, PRKD1, PGM1, SIDT2, MDM4, JAZF1, MET, C3orf58, USP31, PER2, CTD-3116E22.4, CNTN2, TAF5, ZNF304, CCDC50, VTI1B, C5orf43, GRM7, KIAA2026, PEA15, RECK, RALGPS2, KDM5D, FKBP1B, DMWD, LRRC7, MIER3, ZC3H4, CNTNAP2, TNRC18, HSPA1B, FAT3, KCNK9, MTA2, COL4A4, IGSF1, PALM2, PLCG1, BNC2, LMBR1L, ARID4A, TMEM200B, CLCN3, RAP1GDS1, GALT, CCL22, FOXN3, FAM175B, SURF4, CCNB1IP1, CASP2, CALCR, AXL, C11orf34, RFX3, DARC, ADAM10, KLRD1, PALLD, ASB1, ACSM2B, PCF11, DYNC1LI1, TSHZ2, ACOX2, CPLX2, CBFB, HTR2C, PLEKHH2, LGI1, NFATC4, MAN2C1, AC006116.20, IRGQ, WASF1, CYP4F3, PPM1A, SAMD12, RAB21, SLCO3A1, SHMT2, GMNC, EPHA4, CACNB3, HMGCL, MUC19, TATDN2, SLC51A, TMTC3, IST1, RTN4RL1, SULT4A1, SNTB2, BTBD18, ZNF831, MARCH8, MAP4K2, TMEM246, PHF19, FBXO30, ARHGAP19, ADD2, RPL37A, MPP2, ANHX, INHBB, CAMK4, PITPNC1, BCL6, RCAN1, SDK2, SFT2D1, TMEM167A, STAC2, AGTR1, TRANK1, TMEM130, RRAGD, ASB4, FOXP1, C9orf35, CCDC85A, TSN, C4orf22, EEF1B2, COL12A1, ACSM2A, LIMD2, AFF4, THUMPD3, ZDHHC23, VWDE, ATRIP, PHF15, TMEM35, UCN2, PLCB1, NR4A2, ANKRD52, LIN54, FAT4, C15orf23, PCLO, GRK6, LOXL3, ARHGDIB, NLRC5, KANSL2, RPGRIP1, PTPRR, ADO, FGF23, AMZ2, SGK1, FOXG1, SERPINE1, CALN1, MBLAC1, AC009113.1, MFHAS1, SIDT1, ZFP41, PLCXD3, CDH9, CNIH2, SLC35E2, C7orf49, NOS1AP, LTBP3, IL6R, SYNJ1, TAC3, CDK18, C5orf42, PIP5K1A, AFF2, ABCD1, PPAPDC1B, TTC19, DPYSL4, C16orf58, RPL32, RALGDS, SHMT1, FNDC3B, ARHGAP26, USP3, FAM22D, DCAF11, XYLB, CNTNAP1, CALB1, ALG3, FUT1, SEPT3, DPP3, NOTCH2, C8orf22, MSANTD3-TMEFF1, ZNF263, BCL2L13, AAGAB, DNAJB1, PDE7B, UBN2, MCOLN2, ANKS1A, KITLG, SGSM2, BSN, JAG1, TUSC5, PCDH1, KCNJ5, GK5, STC1, SLITRK4, TMEM164, ZNF285, PREB, FAM176C, ZHX3, PAX5, SPEN, PDCD4, LYPLAL1, FOSL1, EML5, ALG6, CLOCK, BEST1, GPR22, TPPP, NAIP, FAM162B, NEU1, HIP1, RFX8, KIAA1217, PLIN4, TWIST2, C6orf222, ATF1, KCNH7, VPS37B, ZYG11B, LMTK3, PPP1R8, SYT5, ACBD3, L3MBTL1, RP11-93B14.6, NPAS4, PRELID2, ARHGEF33, ELL2, LDOC1L, HMGN4, RTF1, PARP8, ADRA1D, MTUS1, RAP1GAP, GLCE, RTTN, CYB5B, PIK3CB, ROCK1, ERBB2, CD47, STX17, ZNF789, NTRK3, WSCD2, NDUFC2, ODZ1, RELN, KRT13, GUCY1A3, PRRG3, CD200, CFLAR, WDR63, CALCOCO2, FAM22G, ENAM, SIRPB1, ZFHX2, RPS6KL1, SSX5, EDAR, GATA3, RPL3, MGAT5B, TUFT1, SEMA4C, PRKCB, PGF, GMFB, SNX12, SAR1A, PPM1L, SLC35G2, BCL11B, GREM2, EFNB1, GYPE, ATP6AP1, C1orf95, SLC4A7, KIAA1210, CCDC88A, CA7, ITGB8, ERICH1, JHDM1D, CTTNBP2NL, MED8, WI2-2118C23.1, EI24, SLC6A1, GFRA1, CPEB2, TCTEX1D1, SHOC2, SDR9C7, CPEB3, MAP7D3, IL1RL1, SNAI1, RORA, FAM83A, SMAD4, GNPDA1, TMEM48, TTLL7, ATG4B, LPCAT3, JAKMIP1, SEMA5B, CUEDC1, C9orf47, OXSR1, OLFML1, ABCB1, CSRNP3, PID1, KIAA0182, HECW2, NAGPA, LILRA1, HS2ST1, SMPD1, DSC3, CHPT1, CTNND1, YTHDC1, SH3PXD2A, VTI1A, GLRA3, ZFP41, TOX, ASAP1, OR10G2, CELF2, PSMD1, SLIT3, TMEM104, PSD3, RASA2, SLC15A5, ZNF593, PTPRD, PRPF38B, PARP15, DYRK4, HMGCS1, GCH1, KIAA1462, POU2F1, WDR96, TMSB10, HNRNPUL2, B3GALNT1, CNTNAP4, CREBRF, PTPN22, ONECUT2, IFI35, TBRG1, F8, PRKCQ, MIR3654, GNAQ, DMPK, RCVRN, COMP, DPF3, ASCC3, CDK6, ORMDL3, ROR1, MAGT1, HLX, RASGRP4, MRVI1, LMAN2L, C22orf39, NISCH, RNF128, THSD4, POMGNT1, KIAA1211L, ZNF148, CHRD, KIT, RIMS3, SOX5, KSR2, SLC6A14, DYNLRB1, POU6F1, KIAA1704, EME1, TMEM25, MMAB, PEG10, FSTL1, UBR1, LMAN1, CLDN18, ZNF275, ZNF16, CDC25A, LRRFIP1, SATB1, AXIN2, TARS2, TPD52L3, DAG1, NRN1, ATG9A, DAB2IP, CDC23, PSD4, GCOM1, YY1, SLC27A4, AMOTL2, TSPAN18, MTMR4, GOLPH3, RP11-59H1.3, MTMR9, KCNG1, TFCP2L1, ZNF282, CORO1C, RP5-1187M17.10, MAP1A, ANGEL1, C15orf53, RP13-279N23.2, IGFBP3, KRT74, NKTR, CELF6, RAB3B, RIMBP2, MDGA1, DNAH3, CDH10, USP43, ESPL1, IGSF3, SYNC, TSEN15, KLRC4-KLRK1, IL9R, DHRS13, AIPL1, BAZ2A, APH1A, SCN1A, MYRIP, DPYD, CHM, TP73, IRF4, PPP6C, ZNF530, CBX3, SDHAF2, FOXR2, LPPR2, KCNQ3, CLIP3, ARSB, USP8, MYOCD, NQO1, XBP1, ATMIN, SERINC1, ZFC3H1, IRF2BP2, MYB, SHPRH, PLN, DBNL, UBE2K, USP24, HSPB6, MAF1, KIAA0825, PLA2G2F, CERS5, PTCHD1, RBP1, THTPA, LPIN3, PTPRM, RAB43, HPSE, USP9X, HTR2A, CHAMP1, RNF170, ATG2A, CITED2, RRAGC, TM9SF3, RAB35, AHCYL2, FYCO1, CSNK2A2, SCUBE3, SFTPC, DYRK2, ZMYND11, AC002472.13, MARCH5, PEAK1, RNF8, PDE7A, HSPA1A, FRMD5, SESTD1, FETUB, FRMD4A, YY1AP1, TMX2, SORBS1, ARGLU1, SNAPC1, XIRP1, RP11-729L2.2, TBC1D30, CSF1R, MYH9, SLC2A13, RPGRIP1L, TBX2, NCOA1, SLC39A10, COG3, SARDH, GPATCH8, GABRB2, UBE2NL, MYO5A, TMEM167B, PTPRB, PTPRT, ZCCHC17, EZH1, LHX2, CFTR, SLC5A3, TMEM133, PNPLA8, GAS8, CLIC5, MIB1, P2RY14, ICOSLG, FGF7, FRMD1, ERLIN1, ATP5SL, MID1, ANP32B, RASGEF1C, QPRT, FKBP9, C10orf128, CASKIN2, ZHX2, EEA1, MFN2, MCIN, DGKI, SNX13, SNX30, MTRF1L, UNC13C, GORASP2, FGF13, GPR6, KLHL23, LCMT2, TMCC3, RP5-977B1.10, NOL8, ALS2CL, BMP8B, C8orf37, NEK9, PARVA, SNED1, ZNF774, RHPN2, MARCKSL1, VPS51, UCP3, DSTYK, ZNF844, LTBP2, C17orf28, ARSJ, C9orf69, VWCE, SRPR, UNC119, TRIM32, ZNF449, SOX4, APOBEC1, NT5C2, CLEC2D, MCFD2, IKBKE, TNFSF14, CHST3, ADAM22, AL360004.1, ZBED3, UBA5, FAM117B, XPO4, MORN4, SLC22A6, ANK3, SYT15, PDSS1, KIF17, ATG2B, PPP3R1, ADCY5, NR1I2, THRA, LRRC55, EIF4EBP2, DGCR6, ANKRD33, HACE1, KIAA1715, EID1, ZNRF3, DAGLA, TMEM52B, NCDN, KRT40, USH2A, NFAT5, NDST1, , ATP8B4, PGRMC2, ALMS1, IL2RB, JPH1, VWC2L, CHMP7, TDRD6, CDKN1C, SPG7, SOCS6, SLC17A3, ZNF502, RDX, PPP4R1, SVOP, THSD7B, DDX58, VPS4A, SH3PXD2B, C16orf5, ERC1, TEP1, HIST1H4H, SIX3, FEM1C, TNKS, EMR2, BBS12, VTCN1, GLS, SLC30A3, TMEM184B, CAV3, HSBP1, NPHP3, FOXP3, STK38L, GJA5, FAM53C, SVIP, UBE2QL1, TTLL1, KRT20, KRT82, PLEKHM1, CBX2, DENND1A, COX7A2, CACNA2D1, RNF169, FANCG, MTMR10, GPR85, SEMA5A, SLC45A3, MRPS14, SERGEF, SCN2A, SRSF11, ARHGAP1, COL2A1, MCTP1, DNM1L, WFDC5, HSPA13, MFAP4, C2orf69, HRK, LAMC1, ZNF672, SPTB, TNFAIP8, CYBRD1, FMNL2, LDHA, SPRY3, SOAT2, KLHDC8B, PDE4B, RDH11, ADAMTSL4, PXDC1, C2CD2, TP53TG3, TP53TG3B, PTGR2, CADM2, UBXN2B, THEM4, TP53TG3C, LIN28B, ECI1, AC133485.1, CD86, USP34, TMOD2, FEZ2, AIRE, ARFGAP2, GLI2, KCNE3, ATG5, C5orf63, LOH12CR2, TPO, UHRF2, EFEMP2, SAP30BP, CAPN9, MAPT, EFNB3, RNF44, MCM2, DNAJC16, NAA30, ZC3H12B, LOXL1, TGFBI, NOP2, PPP1R3E, CCNJL, RNF213, PROX2, DDX59, ANKRD17, GP5, IL17RC, NDRG1, LPP, STK36, CELSR3, MAOA, CYFIP1, GTF3C4, TGFB1I1, IRAK4, ZNF512B, ST8SIA3, AR, NDUFA9, KCNA1, HNF4G, ZBTB40, LENG8, RARB, ZBTB46, ATPAF1, UBL4A, PODXL, SOX6, REV1, FAM40B, SCP2, MLLT4, MAPK13, PHKB, CSNK1G3, TMEM79, LCT, LIN28A, PKIA, FAM127B, TMEFF1, VWA3A, ACOT8, TRAFD1, CACNB1, HPCAL4, STARD8, IRAK3, SLC7A6OS, NAA50, CCR1, C9orf152, C2orf71, SPCS2, PEX5L, TXNIP, HTRA1, WDR91, VPS39, C21orf67, FAM46A, NMUR1, COPZ1, PRICKLE2, LZTS2, MYO15A, KIAA1644, TRIM41, SLC17A4, HBS1L, SCARF1, SYNGR2, ODZ4, PRR23B, SRC, DLEC1, C11orf54, COPS7B, MYPN, AKAP13, TMPRSS15, ABR, C3orf70, RP11-169F17.1, HIST4H4, GABARAPL2, TMEM206, EPSTI1, C15orf54, ILDR1, ZNF561, TGFA, FGD3, LTA4H, TOB2, FAM107A, |
| hsa-miR-574-5p | DGKG, AC084121.16, RP11-1118M6.1, NCDN, SPOCK3, RFX4, CCDC91, TM4SF19, SSX2B, PIGB, VSNL1, ZSWIM1, FOXN3, SLITRK3, C21orf88, TGM2, TMEM106A, KCNIP3, TBR1, SHISA9, FBXL20, CCDC88C, ZHX1, CD40LG, CDH12, C1orf173, NMNAT2, PCGF3, TRIOBP, MS4A7, CD82, EHD3, CALCOCO1, C6orf136, ELFN1, C10orf71, DAZL, EDN1, NLGN4Y, NRN1, CD96, RNF19A, LHFPL5, ARHGEF9, HLTF, PTCHD1, FAM83C, MIS12, UGT1A7, MORF4L1, ITGA11, INSL4, ECE2, CBFB, TCF20, SOX6, PAM, HEATR1, SSX5, GALNT3, HCN3, EIF4E2, FSHB, SEPT6, STAG3L3, TNS4, GLRA2, CCDC70, CTSL1, RSPO1, IGDCC4, HHIP, NLGN3, KIR3DL1, SAMD9L, FAM86B2, MCM8, WHSC1, DHX40, ZNF589, C15orf57, SBK1, MKRN1, TMEM150C, KIR2DL3, GAB1, HOXC6, ZNF596, ATG16L2, FAM123B, IGFL1, NCAM1, KCNS2, TEAD1, PAPPA2, OLR1, LCOR, ZBTB16, PHF20L1, EP300, KIR2DL1, SRD5A3, CAPSL, ZNF559, TNFSF14, NR2E1, GRIA3, DCAKD, USP54, SLFN5, FAM84A, BNC2, CYP4F11, SEPT3, LYRM7, PPP1R16B, STXBP4, NPC1L1, CCBE1, AHCYL1, SCGB2A2, ESCO2, KIR3DX1, SNAI3, IREB2, LEPREL1, NT5C3, SLC9A1, DLG2, GDF7, PRKACB, BDH1, CCK, FAM104B, PPWD1, FAM86C1, BCAS1, AIMP1, KIAA0895L, GPBP1L1, C7, GLP1R, SSX7, MKLN1, KLF7, PCGF5, C3orf35, PNO1, ST6GAL2, CYP2S1, HPS3, AF127577.1, HSF2, LGI3, BATF2, FOXI2, TCTE1, ZNF738, EDA2R, ZNF337, LHFP, CS, FLG, GALK2, NEBL, RIF1, RNASEL, PRTFDC1, CCDC135, AC004017.1, CLIC1, FLNC, AGPAT2, NCR3LG1, HLA-DOA, SLC26A4, C3orf72, INHBA, MPEG1, SLCO4C1, CNTNAP5, ZMIZ1, CACNA2D2, SRGAP2, FAM19A2, EFCAB5, ELMO2, XRCC1, EIF2AK2, VWA5B2, AATK, UGT8, ENPP5, ACOT13, HOXC8, IL1RL1, SETD2, LARP6, KIR2DL4, C6orf195, ATCAY, G3BP2, TRIB1, SFSWAP, CA10, PFKFB4, ZEB1, PHF16, FAM117B, AFF3, PTBP2, CYLC2, TRIM68, RDH8, SSX3, ATRN, SNAP29, NOS1, AC092171.1, ELOVL6, LRRC28, MEF2C, GOLIM4, ZNF141, UBLCP1, GPR83, SYT3, FGF18, SSX9, FLRT2, TRIB2, PTHLH, RNF114, IGLON5, TBC1D5, NR3C2, CHRNG, CTSE, AL353698.1, CLDN10, RAG1, AC110781.3, BEAN1, GIMAP6, RIMKLA, FMO2, AP1G1, AC108938.5, ACP2, PPIC, SSX7, SMPD1, SLCO3A1, SPA17, TNNI1, ZNF382, KIAA1244, TTC30B, STPG2, RUNX1T1, MMP16, PURA, FMO6P, BAI2, CEP192, TNFSF9, C3orf56, SLC10A7, MAF, CISD3, DCAF16, TRAPPC4, CD84, PDE8A, POLR1D, CLIC6, GTPBP10, FOXN1, NDUFB5, GREB1L, TPPP2, PPP2R5C, CPM, PDCD5, PDE1A, SLC13A1, ROCK2, PRKCQ, ACE2, BIN2, PRSS12, REL, KIR3DL2, C1orf180, PORCN, THRB, ASAH2C, EFHC2, UNC80, ZDHHC14, DNAJC6, NPHS2, CIRBP, RP11-58C22.1, RP1-177G6.2, TRADD, ASAH2, KLF8, S100A12, SPAG6, SLC40A1, RP11-247C2.2, NSD1, ANK1, BMP1, ABTB2, ARID5B, GRIA4, TP53I11, RALGAPB, WDR19, SNAP23, TFRC, PARK2, MPP2, ABLIM1, TIPARP, SLC43A3, EIF2B1, DOK6, SNAP25, SEZ6L, NFIC, ZNF343, TUBG1, H1FNT, PIGZ, OTUB2, FAM81B, RIMBP2, GYPA, ZNF236, CARD10, SNX3, TMEM130, CD22, KLC4, SIGMAR1, TSHZ3, ACTR3C, FAM180A, CSMD2, PLAC1L, GTF3C2, TAC4, SSX1, NACC1, RASSF9, C8orf49, OLA1, JMY, NPEPPS, JMJD1C, NIPBL, CXorf1, COG3, BRWD3, POU4F2, BCL2, EPHA3, AMIGO1, TSNARE1, KIF1B, ZNF275, ASAH2B, C20orf112, BLOC1S6, DDB1, UTS2, HADH, CNOT1, SH3BP1, FIGN, CACNB4, C15orf41, TSEN15, PDE10A, SLC5A7, CXorf36, SLC30A9, C1orf94, ETV3, TTLL6, NKAIN2, UCK1, PLCZ1, CR381653.1, ANO8, MEF2D, SEMA7A, DMD, RAB7A, CCDC115, MLXIP, PSAPL1, MRPL17, INPP4B, FAM110C, TAF4B, CHST11, RAB3B, E2F8, ERP27, CHD6, CLCA4, ZCCHC2, LHX4, AL020996.1, DDR1, BICD1, DCAF8, NCK2, AGPAT4, METTL13, FPGT-TNNI3K, CTDNEP1, RNF168, CCDC6, RP11-276H1.3, VWA3A, LMBRD2, CD83, AL158821.1, EFHC1, ANKRD30B, AKT3, ZZZ3, RAPGEF4, ZNF134, LRBA, H2AFJ, MED26, SMAD9, COL1A1, FAM60A, CHRDL1, PCLO, VIM, ZC3H13, GRID1, C12orf39, ONECUT2, DEDD2, GIMAP1, ASAH1, CLIP4, SYTL4, EXOC4, NFAT5, FNIP1, NFKBIZ, ATP2B2, TMX2, CCDC171, FKBP10, KCNC3, MUC4, SIM1, KIAA1737, ASXL2, ZNF706, TMTC1, ZNF618, FNTA, NKD1, GFPT1, POLE4, C6orf170, SLC7A5, FBRSL1, IGFBP7, KIAA0040, CNTN3, ALDH3A2, TREML2, MLLT3, VAMP4, RIMS1, SLC25A43, F9, RNF217, CYBB, HOXD9, RALGPS1, TTC22, NR4A2, TIRAP, MORN5, MPP5, LCORL, JAM3, KATNAL1, RHOJ, RPH3A, CIITA, TXNDC5, C5orf62, FLG2, FAM193B, SLC31A1, FAM86B1, RND3, LYVE1, DNAJB1, KIAA1958, CA4, VWC2L, FAHD2A, GPR180, GOLGB1, ATMIN, DAB2IP, KMO, CDNF, FOXJ3, GJA3, PIK3C2A, USP37, AC005035.1, CHMP1B, RP11-169F17.1, STXBP5L, MGRN1, ALS2CL, WAS, VKORC1L1, BTLA, SLC23A3, FAM18A, ADAMTS15, PHKA1, REEP1, LCLAT1, MRVI1, BVES, AP000867.1, DNAJC9-AS1, SPDYE1, GAPVD1, PSMD9, LUZP4, ZNF772, CA5B, PLEKHG3, PLCB3, CYP19A1, RAB10, GOLGA1, SOX2, SLC9A9, PELI1, ADAMTS12, PDGFC, XPO7, COBLL1, PAX4, NR1D2, AIG1, EEF2K, S100A7A, ZBTB7C, NSUN5, REV1, CACNA1E, IQCJ, ARID3A, CEP89, EYS, ZRANB3, GCSAM, EBF4, MARC2, SYT17, PGM2L1, NTRK2, SOX5, ZNF746, TSHR, GNPDA1, PCCB, RP11-422N16.3, PRSS8, PDGFB, TMEM178B, GKN2, G6PC2, COL4A6, METTL9, AFF2, ATRIP, STK17A, MALT1, LIMS3, CREBZF, KLHL6, FOXO3, ZNF668, LRRC16A, MEFV, ABR, PHKA2, ZMYND8, C2orf72, KAAG1, IL2RA, NAB2, RAB5B, CDKN2B, TIMP2, WNK3, MAP1B, AXL, APOBEC3D, EZH1, VAPB, SLCO5A1, MED6, RP11-826N14.2, AC012215.1, LRRC10B, SLC7A1, PHF6, MAPK10, ECM1, ANO4, LEPR, GLYATL3, RP11-872D17.8, RPS17, CXCR5, IDH3A, SAMD9, RP11-697E2.6, PIK3IP1, CDC42BPB, SNX33, DOCK1, WASF1, ABL2, MNT, AKNAD1, C20orf202, CNOT10, FAM5B, FTSJD1, TCF12, SLC22A4, SFXN1, LYPLAL1, NFASC, CXorf64, KIDINS220, |
| hsa-miR-129-2-3p | TMEM136, VTI1A, ARAP2, AL139147.1, CCNT1, BZW1, MAP3K1, DOK4, NUCKS1, MMP16, LIN7A, GPRIN2, SACS, CAMTA2, AHCYL1, CCP110, H3F3B, BAALC, ZNF233, SNX13, TMEM33, DGKI, NISCH, IL17A, FRYL, FAM206A, CLEC1B, KPNA4, BX255923.1, RIMS3, NMNAT2, STX6, DPY30, XKR4, PTAR1, UNK, VSTM2A, LHFPL2, HEY1, KLHL13, NAV3, PTGER4, DYRK3, NFATC2IP, ZNRF3, FAM210B, FAM120AOS, HMGCS1, SUPT7L, YTHDC2, ELOVL2, SPATA2, PBLD, TIAM1, DYNLL2, PPP6C, GPR137C, VEZT, C14orf28, FRS2, SZRD1, ABLIM3, ANKDD1A, MAP2K6, CGGBP1, PPWD1, DCX, BAG3, RCAN2, NPTN, BSND, JPH4, RTN3, USP25, RNF8, UBR7, RAB8B, THEMIS, GLRA2, SYTL5, RRM2B, RHBG, KCNH6, ACTR6, KIAA0182, KIAA1217, COBL, CDC42BPG, CDCA7L, USP13, BMP1, SUFU, SOX4, KIDINS220, COQ3, HSPH1, MYO1B, VANGL2, EXPH5, BCL2L2, MYCBPAP, HIBADH, FNDC5, ALKBH1, DOLPP1, CASC3, SRPK1, CALN1, PAIP1, LZTS1, KCNK9, MCU, ACADM, NUP98, ELOVL1, GBP5, DEDD, CHST14, C10orf47, STARD4, KDM3B, RCHY1, PHTF2, APH1B, WDFY4, PDRG1, HS1BP3, AGPAT4, FPR3, MAP2K4, IFITM10, GPC1, PRKCE, DCUN1D3, ARRB1, RGS9BP, C10orf118, PNISR, LRP8, PTPN1, TPRX1, WWP1, RP11-51F16.8, BICC1, ZNF436, RNF11, FZD10, GLT25D2, GOLPH3L, CAMKK2, RHOBTB1, PLN, TSNAX, TRIB1, SLC18A1, KIAA0226L, ENOSF1, C22orf24, GFRA1, COPS5, CA7, AL583828.1, RFX3, SCN3B, CACNG8, RREB1, C17orf75, PRKAB2, CALCOCO2, MPZL1, KIAA0430, ZBTB34, SECISBP2, ZNF507, PIK3IP1, NR6A1, YIPF3, FAM8A1, C11orf9, SLC35G3, C2orf88, KIAA1024, MARK2, AC006455.1, ACAP2, NFAT5, ACACA, RIC3, RAB31, TMEM189-UBE2V1, ZNF740, PLXNC1, ZNF879, CRTC2, EPN2, CYP19A1, CHRNA2, SNX15, RGS16, ITGB2, RHOU, ZBTB38, SESTD1, LRP6, ZBTB44, TOMM20, SLAIN2, LPAR3, PKN2, TXLNG, IL17D, STIM2, FER, TRAF4, SEC14L1, AMFR, TBC1D22B, PHF21A, MAFG, SCNN1A, TCHP, PRKCB, GALNTL5, RBMS3, TPO, OSBPL10, RMND5A, RILPL1, ELOVL6, VSIG10L, STT3B, MRPL35, ZFAND6, RAP1B, PAK2, PREX2, AIF1L, TMED8, WDR89, PABPN1, AGPS, CEP63, GNA13, HECTD1, ITPR3, ANKRD52, MSL1, L3MBTL1, MSH6, SCNM1, USP6, C14orf101, CELF4, BIRC6, TGOLN2, ESPL1, NFE2L1, FTO, POLK, PARP1, COL24A1, CACNA1E, TLR10, CNOT6L, GRAMD2, FAM69A, GOLGA8IP, SELV, CA5B, EIF3J, RAB3GAP2, ZNF639, AK4, PPARGC1B, ZNF609, PPIL2, LTA4H, PDLIM2, C3orf33, BCL7A, MRPS25, HNRNPU, RASAL2, CXorf1, RPAP1, CDH20, QSOX1, SAYSD1, GABRA1, DCAF7, ZNF200, SLC25A29, COX4I1, ZCRB1, CNOT6, RALGAPB, SF3B3, GJA1, ZMAT3, C17orf49, NFAM1, LTBP3, DBN1, BORA, SHF, ZNF207, SH3RF1, EMD, NXPH1, LCOR, KL, PTPN3, TMF1, MLL3, CERS3, VPS39, HEATR6, SEC63, TRPM3, CDK5R1, TPRG1, PTPRT, FAM65A, PPP1R14C, RP11-702L15.2, NSD1, TDRD6, KIF24, MAP3K3, PIKFYVE, GRASP, ACSL1, TM9SF3, PA2G4, TGM6, TXLNB, FAM220A, CSRNP1, FAM124A, N4BP1, ANK1, TRHDE, CRHR1, CCNJ, FBXL17, FAM105B, TBX2, SHISA6, PRKAR2A, TNNI1, ZBTB8B, PSAPL1, RICTOR, ERC2, RAP2A, ELFN2, ABCC5, ZDHHC9, ENTPD3, FMNL3, CFLAR, CNTNAP1, TRIM27, EXOC7, S100Z, CEP78, C1orf95, CEP104, KRT35, SMNDC1, ANKRD18A, MAPK3, CDHR1, SCD5, ZNF292, MFSD9, PLCE1, CXCL6, ZHX3, C9orf69, SBK1, MECP2, HFM1, PTPN20B, CHML, CCDC99, POTEC, C1orf141, RBM43, PHC2, YES1, ROBO1, CSRP1, SNX6, BNIP3L, GRAMD1B, NXF1, MEF2A, RPL27A, PKIA, BRK1, PDE10A, TMEM100, STK4, ZNF648, RP11-664D7.4, MUC21, SSH3, RMND5B, RNF175, FBXO36, ASIC1, ADAM22, PIGS, BDKRB2, C12orf76, TEX14, DNPEP, ERLIN2, PIK3AP1, PORCN, KLHL20, TANC2, EPB41L4B, RAB5B, AC009237.1, SLITRK5, AC010642.1, WBSCR17, ZNF229, NCAPD2, E2F5, FMNL2, HPS4, KHK, ADARB1, CCDC47, TP53INP2, MKI67, RP1-130H16.18, C14orf43, NCR3LG1, ZNF451, APBA1, SYNJ2BP, RNF34, PDK2, NLN, BEST2, CSPP1, GRIK3, BRE, |
| Hsa-let-7b-3p | SLC35A3, PLAG1, PUM2, INO80D, ZFPM2, MAST4, PSD3, PDS5B, EBF3, GNAQ, LRP6, ARID4B, TAOK1, COL12A1, C11orf58, REV3L, ATRX, PPP4R2, NRXN1, CALM2, ZMYM4, RAB11FIP2, PPM1L, SLC44A1, TSHZ3, HMGXB4, WWC1, CUL5, KLF12, PRKG1, PNISR, GRM3, NEUROG2, LSM14A, KIAA2018, RAB2A, CRISPLD1, LCOR, ICK, ACVR1, HHIP, MED14, IER5, YY1, FZD3, NFYB, KIF5C, LRRC16A, NR3C1, PIK3C2A, RAB10, OTUD4, ANO4, VCL, CCDC126, AL353698.1, SRSF11, FBXO33, MARK1, PCDH8, RTN1, ANKS1B, MAP2, MED13L, VCAM1, DAGLA, PAFAH1B1, PTMS, HMGB1, PTBP3, PARD6B, SLC18A2, ALDH1L2, CNOT6L, AHR, GSK3B, KIAA0240, SYNCRIP, RBBP7, ZNF720, ZBTB44, MEF2C, SPOCK3, LNPEP, TRIM63, ADIPOR2, MYCN, NBEA, PTCH1, LIPG, RPRD1A, SOX5, GTF2A1, ATG2B, GOLIM4, NRIP1, PUM1, HCFC2, ITGAV, CPEB2, DCAF7, PPM1A, ARAP2, SMEK2, ZC3H11A, PHACTR2, DKK3, ZNF85, ELAVL4, ELOVL2, UNC79, BDNF, CUX2, HOXA9, IREB2, STK40, APP, C10orf118, ULBP1, NR2F2, FOXO1, NLGN1, BTNL9, TRIM61, PDE5A, LCORL, CREB1, BMPR1B, NDFIP1, CASK, GTF2I, STOX2, NCKAP1, NCAM2, ZMYND8, JAG1, SMARCA5, KLF5, FRS2, PAN3, HDAC9, SOX9, FYTTD1, RSPO2, PASK, MMS22L, MYCBP, FOSL2, ARID5B, HMGCR, MLL3, SNX31, BMPR2, HOMER2, ZSWIM6, FAM19A1, TAS2R14, PEX5L, SOX2, PHYHIPL, WDFY3, FAM18B2, DMTF1, GRM5, SPRY4, EMP2, CXXC4, ENSA, ARID4A, HOMER1, CRK, USP6, PTGFR, VCAN, SPRED1, DCUN1D4, SF3B1, WAC, LGR4, ARHGAP20, KIAA0907, NCR3LG1, MARCH6, POLR2K, CBLN4, MARCKS, SNAI2, FAM105A, EPS8, ZNF585A, MEX3B, ELK4, MOSPD2, USP25, NUDT4, FZD5, DCLRE1B, PAX6, FAM76B, ZNF236, KLF6, AC079354.1, RHOT1, TFRC, CDK19, DOCK1, KDM6A, GRAMD4, IRS2, KAT6B, JUP, UBN1, LRRC8B, EGFL6, SIX3, MYCBP2, PELI1, PPP1R3F, BASP1, HMGN2, MOB4, CTD-2368P22.1, SCN2A, NHS, FAM18B1, NFAT5, TADA1, WASL, CLDN1, ZNF800, POM121L12, ZNF827, SORBS1, ACTR3, ZNF384, SMO, NUP153, FBXO34, OPA1, SET, SMOC1, IKZF2, HNRNPA1, ESRRG, PTER, EXOC6B, GATA3, UBR5, KCTD12, MAP3K2, MYT1L, ERBB2IP, SNAI1, GLCCI1, ZNF207, KANSL2, CCL7, IRF2BP2, PEAK1, CCNY, PSPC1, ANKHD1, DMXL2, EPC1, LRRC16B, C5orf30, MALT1, SETX, FAM126A, RAP1B, ASAP2, UBE2W, MBNL1, FAM107B, BAZ1B, EVX2, SSBP2, NCOA2, EFNB2, AP1AR, PTAR1, WDR48, HEY1, RNF149, C1D, SP2, CENPC1, GTF3C3, USP7, PRKRIR, PROX1, MPHOSPH9, BMP3, SOCS6, UBN2, MBD5, RBPJ, NUP160, KIF3A, NFIC, FAM3C, FIGN, NTRK3, UBR1, TOB1, ZNRF3, FGF13, SIX4, XPO7, ELAVL2, HNRNPA2B1, XPR1, ID4, KIAA1217, NRP2, JHDM1D, DAAM1, CNR1, ARRDC3, TPD52, SKIL, MAPKAPK5, FGD4, RALGDS, RNF34, SRSF12, TTC14, CNBP, TRIM23, VCPIP1, FRMD4B, EEA1, PIK3C3, GALNT7, C16orf52, GIGYF2, SLC2A13, RBFOX2, ECT2, LRRC7, MAN1A2, PCDH19, STRBP, BRD1, VEZF1, ITGA6, NR5A2, SERP1, RAD21, RAB14, DEK, SAMD12, CASD1, PRR12, PPP3CA, MN1, USP6NL, CEPT1, DOCK11, LYSMD3, ADNP, ERAP1, CNOT6, CACNA2D1, JPH1, SIRPA, BTBD7, HCN4, ATXN1, LIN9, PI15, ART3, ARHGAP12, RBM27, PPP1R2, IQSEC2, KIF11, ID1, RP11-386G21.1, CEP78, SSX2IP, STXBP5L, RAB33A, KIAA0408, ARHGEF33, ARFGEF2, GABRA1, CRTC3, MAP4K3, ATF2, TMTC2, FSD1L, DNALI1, KHDRBS3, HIPK2, CCNA2, RNF214, FNIP1, CPNE2, ATG12, ZNF652, COL14A1, HMGB2, NAA20, RBM12B, SLITRK3, SALL1, EGLN1, SLC34A2, LRP2, PAPOLG, TFAP2B, KIN, CHD9, ZNF292, VAPA, CNKSR2, TTBK2, CLOCK, ZNF436, TCF7L2, C7orf73, ATPBD4, USP12, DENND4A, PRDM2, SOX21, RNF152, JAK2, MAP2K4, DAZ4, DNM3, ARL5A, APLF, FUBP3, GPR158, ITCH, PRDM12, EIF4A2, HIP1, OXR1, RGMB, VMA21, ATF7IP, CNKSR3, PLXNC1, ARGLU1, RNF2, CNTN1, LRP1B, PCSK2, ASXL2, RHOA, AMMECR1, EIF1AX, TTF2, WDHD1, GDAP2, JAG2, ALKBH8, EFNB1, ZNF430, LMX1A, NTN4, LAMTOR3, KCNJ3, F3, SEMA3C, MLLT10, RSF1, SCAF8, PHACTR4, MAN1A1, CEP41, DDX21, PRPF38B, CAMTA1, RNF38, WASF3, RALA, SLC39A10, LRRC58, CDC14B, AFF4, RP11-322L20.1, MSTN, ROCK1, TMEM192, CUL1, KLF3, ASXL1, EIF2S3L, RFX3, TMX4, CLDN12, NUFIP2, KIAA1468, GUCY1B3, ULK2, ZBTB39, REPS2, TSC22D1, GABPB2, CNTN5, MED6, LATS1, RNF216, KIF14, HNRNPD, MRPL44, RP11-884K10.5, ENOX1, PECR, SEPHS1, RASEF, CDK17, IGFBP1, SORCS3, CTDSPL2, RASGEF1A, PFKFB3, RIMKLB, RB1CC1, FAM9C, BTAF1, ST6GAL2, COL4A3BP, TOR1AIP2, TBC1D15, DKK2, ATRN, CPEB3, COBLL1, LEMD3, ID2, MAML2, FGFR2, MLLT6, ZC3H4, EFNA5, FAR1, MED12, MEIS1, MTSS1, PRDM16, CAPN7, TEX9, BRWD3, PKN2, PLEKHG1, NAA25, DCK, CREBZF, UTP20, COL4A1, EFR3A, TCF20, EDEM3, EIF4G3, SLAIN2, GRIA3, SEC22C, NPR3, FAM9A, FRMD4A, FYB, PLXNA4, EPB41L1, POU2F1, ODZ1, TEX30, AKTIP, CDK6, KBTBD2, ANKRD17, SGK3, CD44, MECOM, PARP8, MMP24, USP24, SPRY2, FBXO45, ZNF521, SMURF2, ITM2B, GCLM, SYT1, GTDC1, RLF, ZNF644, ZNF273, SLC19A2, PRPF8, FBN1, SNCAIP, RAB11B, PPAP2B, SLC30A4, HECTD2, CREBRF, UBE3C, NUP107, FNDC3B, RAB3GAP2, KLHDC2, ZMIZ1, ARHGAP15, KIF20B, SEZ6L, RNF19A, HAS2, AASDHPPT, LIFR, FOXN2, NCAM1, C1orf27, AKAP10, ZFP28, LRCH2, AXIN2, ALS2CR8, ABI3BP, INSM1, CNIH, UNK, MECP2, SLIT2, RNF217, TEK, HECTD1, ZNF506, TES, CTR9, AGTR1, PTCHD1, ZNF238, MFSD4, RNF144A, NRBF2, ATP2A2, TET2, CNNM4, ZNF738, KIAA1109, TRIM6, MCM9, CXorf22, STARD4, UBXN7, JPH3, PNN, IPO7, PDLIM5, TUBGCP5, SLC4A4, DENND2C, DACT1, ASB11, KCNC2, EIF1AD, PAK2, DSG2, CLNK, ZBTB49, SPTSSA, PHF20L1, RSL24D1, SPAST, RASGRP1, TTLL5, NKTR, RFX1, CHEK1, NIPBL, SOCS4, TRPM7, TGFBR1, MORC3, IMPAD1, IFNAR2, POU6F2, COL11A1, CEP44, SRSF1, C1orf63, MET, USP1, DCUN1D3, STYX, STK17B, BMPER, FRMD3, SEPT9, PATL1, CLIC4, ZKSCAN1, SPRY1, DPP8, CDC7, KALRN, CDC42, EYA4, RBAK, B3GALTL, TMED7, FSCN1, B3GALT2, DCBLD2, WDR37, C20orf197, CTTNBP2, TSC22D2, TXNRD3NB, GRK5, MSRB3, OLFML2B, AAED1, TSPAN12, TBR1, GOLGA1, PRKAA1, ZNF566, LHX5, PHLPP1, CCDC144A, STK24, HNMT, HHLA2, ZNF12, SPINK1, CLASP2, RRP15, ACSL3, ANTXR2, PPP1R10, PSIP1, MAGI1, EIF2AK2, SORT1, ERBB4, DLX2, SOX11, PRRC2A, MYLK3, TIMP3, TMTC3, GPR116, COL1A2, LMO3, ELL2, TMEM170B, DLG2, ABCA5, DENR, ONECUT2, POU4F2, SLC25A53, SMNDC1, IRX3, TM9SF3, GABRG1, TTC33, TCERG1L, KLF10, FAM117A, PBRM1, LHCGR, SLC5A12, CCNL1, DNAJB5, NFIA, ERF, AFF3, N4BP2, NAA30, CTNNB1, ARPP19, AL137145.1, POC1B-GALNT4, TNS3, TMEM132B, EML1, FAM19A5, NKX2-8, FAM153A, TPM1, NFASC, AC144568.2, C10orf107, WNK3, KIAA0947, PNRC1, SLC23A2, MAP2K1, NSD1, TMEM196, PHF3, FAM43A, UBE2F, SRSF2, KATNAL1, POU4F1, CAPZA2, FGF7, C5orf24, RYBP, CAND1, CLIP1, KCNQ3, CAB39, DUSP19, KIF13A, DTWD2, ASAH1, AKD1, C10orf88, BCL6, PPIG, ADCYAP1, KCNE4, ETS2, LY75, HSP90B1, CDC73, MYO9A, PPP2R5E, EXT1, NHLRC2, GDAP1, CREBBP, CSMD2, EXOSC9, WTAP, HVCN1, HOXA13, RBMXL1, PARP6, STK38L, SPESP1, FMNL2, IAPP, ADRB2, XIAP, CALCR, RABGAP1, NDST3, KLHL2, ZMAT1, YAF2, ABCD2, ABCA1, LRR1, FNDC3A, TMEM56, EZR, HSP90AB1, GP1BA, NLE1, MOB1B, GRM8, PTBP2, CWC22, OTUD3, GPR6, AP1S3, SEMA3D, DYNC1LI2, KIAA0355, CDC14A, WNT5A, ZBTB10, TCF3, TMEM68, NNT, OSBPL8, RAB3B, DSTYK, AUTS2, ERCC6L2, IL12B, FAM201A, NLGN4X, ZNF326, TDG, CHD2, SUMO1, MFN1, SCXA, EREG, DPM1, KIAA1715, PLCB4, FRMD6, EPHA3, FSTL5, DCLK3, GTF3C4, FOXF1, GPR37, LRPPRC, LLPH, FGF16, SENP6, LRRC55, SEC24A, NXT2, DAPP1, KIAA0494, IMPACT, ELOVL7, WDR75, AC091435.1, MCU, ACAP2, BTG1, PDE10A, RRAS2, KIAA0895, SPEN, SLC38A2, GPCPD1, TANC2, KPNA3, KCNE3, CD3G, RAB23, DIP2B, DACH1, ETV3, TNRC6A, FAM123A, TBC1D4, WDR26, ZNF518B, GTPBP10, FBXO43, NPY, SUGT1P3, PAQR3, BHLHE40, SIPA1L2, SLC35E1, FOXP2, PMAIP1, TAF4B, RPRD1B, CFTR, SUSD3, APPBP2, SLAMF1, RAP1GAP, KANK4, C14orf37, NOVA1, WDR72, SMARCB1, DOCK3, ZNF148, ARNTL, C11orf72, SMAD4, AC007431.1, C10orf126, ANTXR1, ZNF501, SRGAP1, MARCH1, UBQLN1, FOXD3, KCNG3, TAB2, BTF3L4, TBCEL, LRRTM4, FAM160B1, ELF2, PANK3, FLT3LG, FAM71D, C1orf204, PSEN2, KCNK2, TRAK2, RAB30, PPP2R2D, RASA1, RABGGTB, MYSM1, GRIN2A, PTPLB, AHDC1, PEX14, NRN1, EIF1, ENTHD1, UNC119B, ZNF532, ZDHHC21, ZNF791, RXFP1, KIAA2022, ANKRD1, SGOL1, MFAP3, ZNF263, ABCA9, MGA, RBMX, SOD1, MBD2, BCKDHB, PTPRO, IPMK, AGFG1, DNAJB14, GABPB1, STRN, GMCL1, TMEM161B, DENND1B, NDEL1, ATXN3, LONRF2, GAS1, IRS1, UBE2C, YTHDC1, NAGK, NEDD4L, CPSF6, MITF, HTR2A, NT5C2, ANO1, PCGF5, MATN2, CSTF2T, CD200R1, SNTG1, COL19A1, SNTB2, MTPN, MPC2, HBP1, SLC16A14, MPP6, TMEM128, TBC1D2B, SPRY3, ITM2C, KIAA1958, ZNF569, ST6GALNAC3, SATB1, NECAB1, NR4A2, B9D1, KCNT2, MAPK8, EDIL3, INO80C, MAK, HIVEP2, CAMLG, EPHA4, NAV3, HSF2, RUFY2, TAOK3, CNTFR, LANCL1, DDHD1, PRPF4B, TWIST1, AC027323.1, WDR25, SH3GL3, XPO1, MBL2, COL8A2, E2F5, FGF2, FAM177A1, C1QTNF7, UBTF, DIMT1, STARD13, ZNF655, TMEM48, LPIN1, CALN1, PIGN, ATP10D, LEPROTL1, CLCN3, UBE2D1, FAM91A1, GIN1, PLSCR1, RNF182, KCNH5, RORA, RCOR1, CCDC132, PRDM1, HNRNPU, PCDH11X, FAM199X, LDLR, ZMYM2, CTLA4, BLOC1S4, ZFY, CTB-50L17.14, ZNF182, SYF2, UBQLN2, MDFIC, FOXO3, PIKFYVE, MDM2, SPIN1, PRPS2, DLL4, ARHGAP29, KIF1B, PTPRE, GABRA6, TMA16, PBX3, CRY1, LRCH1, SIM1, KIAA1841, ATP2B1, NTNG1, PDE1C, GALNT4, BET1, AFF1, PPP1R1C, MEF2A, TNFRSF11B, OPRK1, PTEN, SLC8A1, CORIN, RIF1, MATR3, RBM5, MYO1E, RP11-108O10.8, PDZRN3, WHSC1L1, MAGT1, LIMS3L, CCDC50, ZNF90, ZNF697, ATXN7, CYB5D1, FMR1, SYNGR1, PPP1CB, CD84, LIMS3, MLL5, MAGOHB, ITGA2, TLR4, ARIH1, PDCL, ADD3, ZBTB2, SLC1A2, HIAT1, ZNF92, MBNL2, GABRB2, FAM208A, RNF44, NLN, TRERF1, ATAD5, FOXF2, ZC3H6, LRAT, GBP4, EFCAB7, ENPP1, ALCAM, BTBD1, GMFB, TRAPPC10, CTTNBP2NL, LAMP2, ATAD1, CYYR1, VPS36, CCDC38, AQP2, NIPAL1, WBP4, IL15RA, WDR70, FAM9B, AFTPH, RRN3, PCDH11Y, KIAA1147, LHX9, ZFAND5, USP46, OTUD6B, GOLPH3, FAM190B, KIF22, FKBP7, C16orf70, HEATR5B, RREB1, RHOBTB3, RPGRIP1L, CCDC120, ALG10B, ACBD5, MZT1, LRRC1, MKLN1, GPR34, SLITRK4, ROCK2, CHMP1B, ACOT11, NOLC1, FGD6, SPICE1, SLC25A3, RRAGD, KIAA1429, NLGN4Y, WHSC2, RXRG, ARNTL2, TFPI, RP11-111M22.2, SFRP2, ARF1, FBXL3, RAB22A, PPP2R5C, ZC3H12B, GAPVD1, FCHSD2, CD46, CRYZ, BLZF1, TXLNG, NEUROD1, TNFRSF10A, TEAD1, ZNF573, FBXO32, ROBO2, ZNF367, NOTCH3, WNK1, G3BP1, HRH1. |
